# A self-assembling surface layer flattens the cytokinetic furrow to aid cell division in an archaeon

**DOI:** 10.1073/pnas.2501044122

**Published:** 2025-06-18

**Authors:** Sherman Foo, Ido Caspy, Alice Cezanne, Tanmay A. M. Bharat, Buzz Baum

**Affiliations:** ^a^Cell Biology Division, Medical Research Council Laboratory of Molecular Biology, Cambridge CB2 0QH, United Kingdom; ^b^Structural Studies Division, Medical Research Council Laboratory of Molecular Biology, Cambridge CB2 0QH, United Kingdom

**Keywords:** archaea, cell division, cytokinesis, S-layer, cell mechanics

## Abstract

The surface layer or “S-layer” is a two-dimensional lattice of proteins that coats a wide range of archaea and bacteria in place of a cell wall or capsular polysaccharides. S-layers are thought to play an important role in chemically and physically insulating cells from the external environment. Here, we show that the integrity of the S-layer in *Sulfolobus acidocaldarius* is maintained as cells grow via a process of self-assembly as SlaA monomers fill gaps in the lattice. Although this lattice which is physically tethered to the membrane might be expected to hinder cell division, we show that the S-layer flattens the membrane at cytokinesis to accelerate ESCRT-III-dependent cell division—and is important for robust, successful cell divisions under conditions of mechanical stress. Taken together, these results define the rules governing S-layer self-assembly and show how a flexible lattice coat that is coupled to the underlying membrane can both provide a cell with mechanical support and help to drive rapid and functionally important changes in cell shape.

## Significance

Cells must be protected from physical insults from the environment. Many bacteria and archaea employ an enveloping, protein-based coat for this purpose. Here, we show that the integrity of this coat in *Sulfolobus acidocaldarius* is maintained by a process of protein self-assembly, which ensures that gaps are filled as they arise. While this coat might be expected to limit the ability of cells to undergo rapid shape changes, here we demonstrate that the deformation of the surface coat accelerates cell division. By showing how the mechanically active division machinery and a passive, protective coat work together to give rise to stable dividing cells, this study shows how cells reconcile what seems like a trade-off between mechanical resilience and flexibility.

Because eukaryotes likely emerged from a merger between a member of the Asgard archaea and an alphaproteobacterial cell that gave rise to mitochondria (1, 2), many of the molecular machines that drive core cell biological processes in eukaryotes have archaeal counterparts (3). For example, both human and *Sulfolobus acidocaldarius* cells use homologous machinery, consisting of ESCRT-III proteins and an AAA-type ATPase Vps4, to drive membrane remodeling during the final stages of cytokinesis (4, 5). At the same time, archaeal cell division is also likely influenced by the unique biophysical aspects of archaeal cellular components, including peculiarities of the archaeal lipid membrane, whose lipids are distinct from those of bacteria and eukaryotes, and the highly glycosylated protein surface layer (S-layer) that fully envelopes most archaeal cells (6–9). Since these aspects of archaeal cell biology are not well understood, here we explore the influence of the S-layer on ESCRT-III-dependent cell division, using *S. acidocaldarius* as a model system.

S-layers are two-dimensional para-crystalline structures composed of S-layer protein monomers which are found on the surface of many prokaryotes (6–10). Although there are notable exceptions, including archaea that use pseudomurein, methanochondroitin, or protein sheaths (8, 10, 11), and archaea which lack an S-layer entirely (12–15), most archaea are enveloped by an S-layer. As the barrier separating an archaeal cell and its external environment, this S-layer serves a plethora of functions in different species: providing mechanical support for the membrane (16, 17); physically and chemically isolating the cell from the external environment (18, 19); protecting cells from predation (20); and facilitating the capture of low concentration nutrients from the environment (21, 22).

*S. acidocaldarius* is a member of the Thermoproteota and is one of the most experimentally tractable archaeal relatives of eukaryotes—making it an ideal model system in which to study different aspects of archaeal cell biology. The structure of the *S. acidocaldarius* S-layer was recently solved (23, 24). This analysis revealed that SlaA forms a pseudocrystalline array that envelopes the cell surface with a p3 symmetry, and is anchored to the membrane via SlaB (23–27). Both SlaA and SlaB proteins are highly glycosylated (24, 28, 29)—a property that has been suggested to play a role in guiding SlaA lattice assembly (24).

In several bacteria and halophilic archaea that possess an S-layer, live imaging experiments have revealed the local insertion of S-layer monomers at the mid-cell during exponential growth, implying a mechanistic link between S-layer biogenesis and cell division in these organisms (30–33). In systems that couple the local insertion of S-layer proteins to membrane remodeling as an aid to cell division, division occurs over an extended period of time, and is driven by the constriction of an FtsZ-based division ring—analogous to the way cell wall synthesis aids cell division in some bacteria and yeast (34–36). This is very different, however, from the situation in *S. acidocaldarius*, which divides rapidly using forces generated by changes in the state and preferred curvature of composite ESCRT-III polymers to rapidly deform and cut its bounding membrane within a few minutes (4, 37).

To investigate the assembly of the S-layer and its role in *S. acidocaldarius* cell division, we have combined molecular genetics, S-layer reconstitution, electron cryomicroscopy (cryo-EM), electron cryotomography (cryo-ET), and high-temperature live cell imaging methods (37). In this way, we demonstrate the ability of S-layer lattices to self-assemble on the surface of live archaeal cells through the extension of the SlaA lattice margins via attachment to both SlaB and a thermopsin-like membrane protein. In addition, we show that the S-layer is maintained as cells grow and expand their surface area via the incorporation of SlaA proteins into gaps in the preexisting lattice. Interestingly, by examining cells with partial SlaA lattices, we also show that the S-layer has a preference for the division bridge of constricting cells and tends to flatten the membrane to which it is bound. Finally, by comparing cytokinesis in control and Δ*sla* mutant cells, we show that the S-layer helps to flatten the membrane on either side of the furrow to accelerate cytokinesis, aiding division, especially in cells subjected to physical perturbation. Taken together, these data shed light on the mechanism of SlaA lattice self-assembly in *S. acidocaldarius* and reveal how the S-layer coat helps to reshape dividing cells to accelerate ESCRT-III-dependent cytokinesis.

## Results

### The Generation and Characterization of S-Layer Mutants of *S. acidocaldarius*.

To investigate the role of the S-layer in *S. acidocaldarius,* we generated mutant strains lacking either *saci2355* (Δ*slaA*), *saci2354* (Δ*slaB)*, or both components of the S-layer (Δ*slaAB*) using established genetic methods (38). The mutant cells were viable and their identity was validated via genotyping the loci involved and by whole genome sequencing (*SI Appendix*, Fig. S1*A*).

**Fig. 1.**
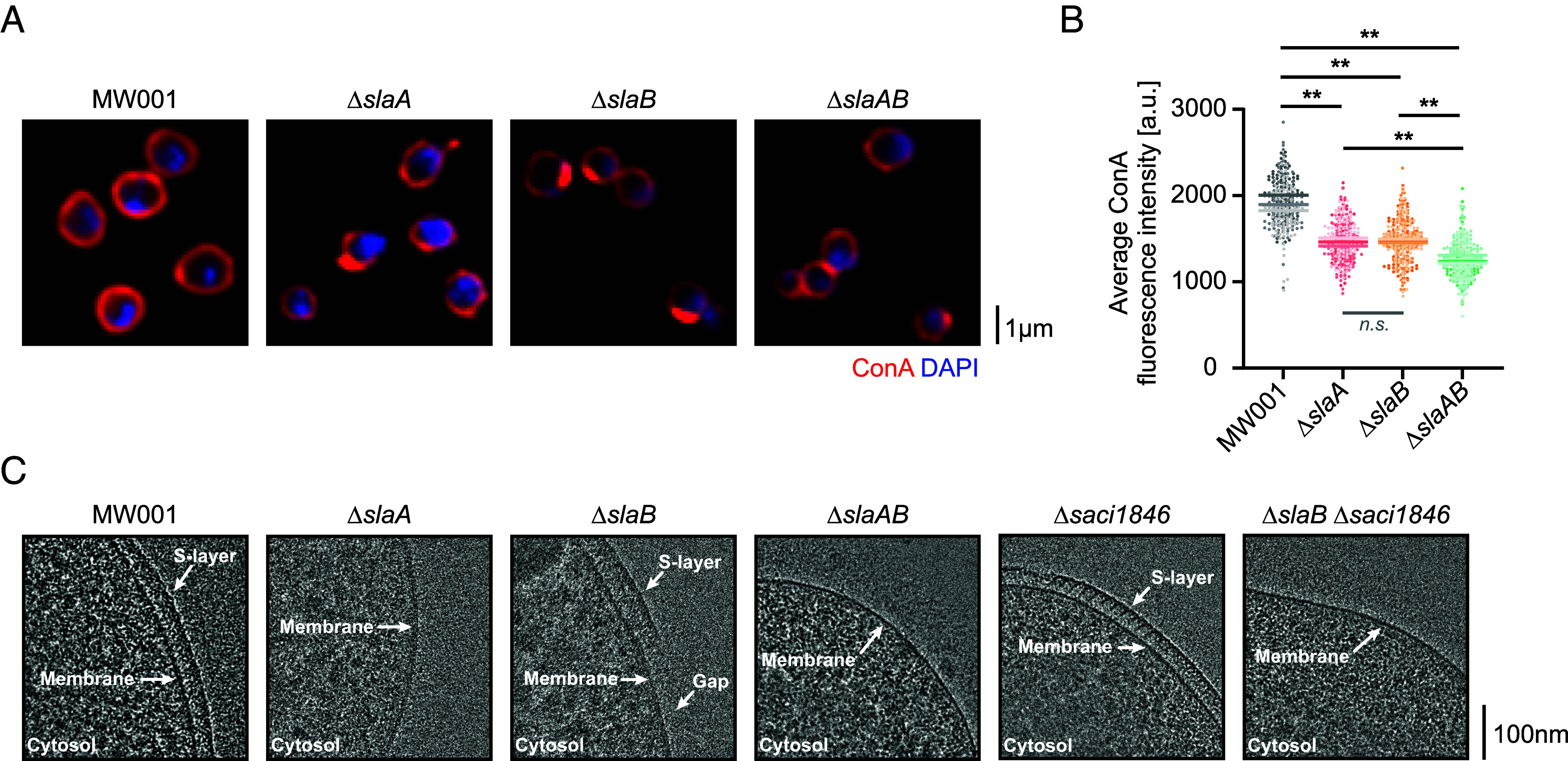
The S-layer mutants of *S. acidocaldarius*. (*A*) Markerless in-frame deletions of *slaA* (*saci2355*) and/or *slaB* (*saci2354*) genes were generated in the uracil auxotrophic strain MW001. Representative single-plane spinning-disk confocal images of these strains stained with the glycosylation marker ConA to visualize the highly glycosylated S-layer and DAPI to visualize DNA are shown. (*B*) Scatter plots of the quantification of the average ConA fluorescence intensity in the strains indicated (N = 3, n = 100 cells each). Each biological replicate is represented by a different shade and the means are indicated (MW001: 1,911.4 ± 71.4 a.u.; Δ*slaA*: 1,465.6 ± 39.5 a.u.; Δ*slaB*: 1,478.7 ± 18.8 a.u.; Δ*slaAB*: 1,260.5 ± 35.9 a.u.). *P*-values were derived using Welch’s *t* test (N = 3; **P ≤0.01; *n.s.*, no significance). (*C*) Representative cryo-EM images of the uracil auxotrophic background strain MW001 cells, the S-layer mutants Δ*slaA,* Δ*slaB,* Δ*slaAB*, and the novel S-layer associated thermopsin like protease protein mutants Δ*saci1846* and Δ*slaB* Δ*saci1846*.

To begin the cell biological analysis of the different mutants, we used fluorescently conjugated concanavalin A (ConA), a lectin that binds to glycosylated proteins (24, 28), to visualize the surface of these cells relative to the control strain (Fig. 1*A*). While levels of ConA fluorescence were substantially reduced in cells lacking either SlaA or SlaB relative to the control (an uracil auxotrophic background strain MW001), and were even lower in the Δ*slaAB* double mutant (Fig. 1 *A* and *B*), ConA was still able to label Δ*slaAB* cells. This shows that, while S-layer proteins make a significant contribution to the total surface glycosylation as expected (24, 28), there are likely a host of additional surface proteins or lipids which are subject to glycosylation that are still accessible to ConA once the S-layer has been removed. We confirmed that SlaA and SlaB are major targets of the glycosylation machinery using mass-spectrometry analysis of a ConA pull-down from the wild-type strain DSM639 (*SI Appendix*, Fig. S1*B*), and by Western blotting ConA pull-downs from extracts of cells expressing HA-tagged variants of SlaA and SlaB (*SI Appendix*, Fig. S1*C*).

### Saci1846 Assists SlaB in Anchoring SlaA to the Cell Surface.

To obtain a higher-resolution view of the surface of SlaA and SlaB mutant *S. acidocaldarius* cells, we next imaged cells using cryo-EM (Fig. 1*C*). In accordance with the recently published stalk (SlaB) and canopy (SlaA) model of the *Sulfolobus* S-layer (23, 24), removal of SlaA resulted in the complete loss of the S-layer (Fig. 1 *C*, *Second* panel). As expected, double mutant Δ*slaAB* cells resembled those of the Δ*slaA* mutant (Fig. 1 *C*, *Fourth* panel). Interestingly however, deletion of the membrane anchor SlaB gave rise to a discontinuous surface coat in which patches of intact SlaA lattices remained (Fig. 1 *C*, *Third* panel). This phenotype is consistent with observations in the SlaB mutant in the related species *Saccharolobus islandicus* and *Saccharolobus solfataricus* (39–41), which led the authors to conclude that the SlaA S-layer peels away in cells that lack support of the membrane anchor SlaB.

Furthermore, this cryo-EM analysis revealed that patches of S-layer remained at a reproducible distance of 37.2 ± 1.3 nm (n = 6 tomograms) from the plasma membrane in cells lacking the membrane anchor SlaB (Fig. 1 *C*, *Third* panel); implying the presence of a redundant membrane anchoring system. In *S. islandicus,* an S-layer associated protein M164_1049 was previously shown to assist SlaB in stabilizing the outermost SlaA layer (39, 41). A pBlast search in *S. acidocaldarius* identified four homologs, Saci1846, Saci1049, Saci1534, and Saci2170. Of these, Saci1846, a thermopsin family protein, proved the closest homolog, being 36.95% identical to M164_1049 over nearly the entire length of the protein (*SI Appendix*, Fig. S1*D*).

An analysis of Saci1846 using Phobius and TMHMM (42, 43) revealed that the protein has a similar overall architecture to SlaB, with an N-terminal signal sequence and a C-terminal transmembrane helix. AlphaFold modeling (44–47) further revealed that, like SlaB, Saci1846 contains three predicted immunoglobulin (Ig)-like domains within its structure (*SI Appendix*, Fig. S1*E*)—domains commonly found in archaeal and bacterial S-layer proteins (9). As the Ig-like domains in the N termini of SlaB trimers were previously predicted to interact with SlaA (24), we performed pairwise structure alignments (48, 49) of the three Ig-like domains of SlaB with those of Saci1846 sequentially starting from the N terminus. The structural similarity was high, with RMSDs of 3.13Å, 3.45Å, and 3.65Å and TM-scores of 0.57, 0.52, and 0.36 for each of their three Ig-like domains respectively.

To test whether the *S. acidocaldarius* homolog of M164_1049 performs a conserved function in stabilizing the S-layer, we generated mutants lacking Saci1846 in an MW001 or in a Δ*slaB* mutant background. Cells lacking Saci1846 exhibited minor defects in the smooth S-layer structure, as visualized using cryo-EM (Fig. 1*C* and *SI Appendix*, Fig. S1*F*). Yet, the S-layer remained anchored at a distance of 36.6 ± 1.8 nm (n = 7 tomograms) from the plasma membrane in the Δ*saci1846* mutant (Fig. 1 *C*, *Fifth* panel). In line with previous reports from *S. islandicus* (39), the Δ*slaB* Δ*saci1846* double deletion mutant resembled the Δ*slaA* mutant in entirely lacking an S-layer (Fig. 1 *C*, *Sixth* panel). Furthermore, trichloroacetic acid precipitation of proteins present in the growth medium of Δ*slaB* and Δ*slaB* Δ*saci1846* cultures (*SI Appendix*, Fig. S1*G*) revealed abundant SlaA. These data indicate that SlaA is anchored at a similar distance of ~37 nm to the membrane via both SlaB and Saci1846 and is lost from the surface of *S. acidocaldarius* cells in their absence.

### S-Layer Biogenesis in *S. acidocaldarius*.

Next, to investigate the dynamics of S-layer biogenesis, we attempted to identify sites of new S-layer insertion. Following optimization and validation of S-layer labeling using Alexa Fluor conjugated N-hydroxysuccinimide (NHS) ester dyes (*SI Appendix*, Fig. S2 *A*–*C*), we performed a pulse–chase experiment to compare the patterns of old and newly assembled S-layer on the cell surface. For this experiment, MW001 cells were first pulse-saturated with an Alexa Fluor 488 NHS ester dye and allowed to grow for 2 h, before chase labeling was performed with an Alexa Fluor 647 NHS ester dye. The cell surface was then examined by fluorescent microscopy. Interestingly, while some label was incorporated at random positions in the surface layer, there was no obvious correlation in the localization of the pulse and chase labeling (*SI Appendix*, Fig. S2*D*), even in dividing cells (*SI Appendix*, Fig. S2 *D*, *Bottom Right*). These data suggest that new S-layer insertion occurs in a distributed manner across the cell surface, likely filling in small gaps in the lattice that are formed as *S. acidocaldarius* cells grow. This is markedly different to reports from other organisms in which S-layer expansion has been proposed to occur in a highly localized manner to facilitate tip growth in *Caulobacter crescentus* and *Corynebacterium glutamicum* (33, 50), or to drive expansion of the midzone in dividing *Haloferax volcanii, C. crescentus,* and *Clostridium difficile* cells (31–33).

### Visualizing the Self-Assembly of the SlaA S-Layer.

**Fig. 2.**
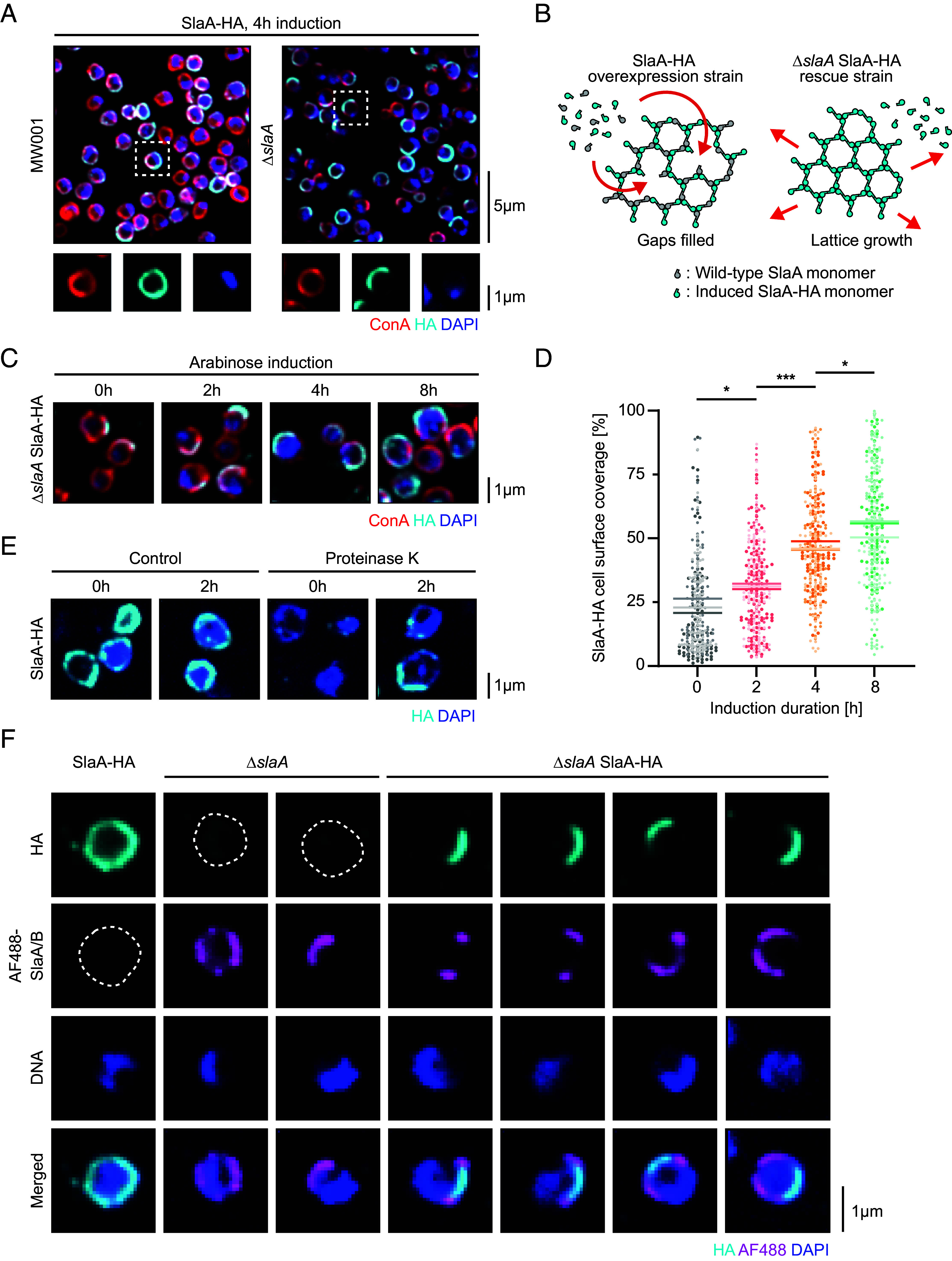
Visualizing S-layer biogenesis and assembly in *S. acidocaldarius*. (*A*) Field of view showing a representative single-plane spinning-disk confocal image of C-terminally HA-tagged SlaA following 4 h of induction in the MW001 background strain (*Left*) or Δ*slaA* deletion mutant (*Right*). Magnified images of a representative cell indicated by the white box in each example are shown below. (*B*) Model of the SlaA S-layer lattice formation in *S. acidocaldarius*. Expression of endogenous (gray) and HA-tagged SlaA (cyan) in the overexpression strain ensures even coverage of the cell surface due to filling in of random gaps that arise in the lattice. In the rescue mutant, the SlaA-HA lattice self-assembles and expands outward from a nucleation point. (*C*) Time course of representative single-plane spinning-disk confocal images of the Δ*slaA* SlaA-HA rescue strain over 8 h of arabinose induction. (*D*) Quantification of the SlaA S-layer coverage in Δ*slaA* SlaA-HA as a percentage of the total cell surface at the middle z-plane over time (N = 3, n = 100 cells each). Each biological replicate is represented by a different shade and the means are indicated (0 h: 23.3 ± 2.4%; 2 h: 31.1 ± 0.90%; 4 h: 46.8 ± 1.5%; 8 h: 54.3 ± 2.8%). *P*-values were derived using Welch’s *t* test (N = 3; *P ≤ 0.05; ***P ≤ 0.001). (*E*) HA-tagged SlaA overexpression strain was treated with Proteinase K and SlaA-HA levels allowed to recover over 2 h. Shown here are representative immunofluorescence images. (*F*) Addition of fluorescently-labeled (Alexa Flour 488, AF488) exogenous S-layer proteins to cell culture medium was performed to identify sites of S-layer growth. Shown here are representative immunofluorescence images of SlaA-HA, Δ*slaA,* and Δ*slaA* SlaA-HA cells following 4 h of arabinose induction and 1 h of incubation with fluorescently labeled exogenous S-layer proteins (AF488-SlaA/B).

As an additional method by which to measure S-layer protein assembly, we designed a C-terminally tagged construct that could be used to express SlaA-HA under the control of an arabinose inducible promoter. Induction of the SlaA-HA expression in MW001 cells led to the seemingly random insertion of newly expressed SlaA-HA into the S-layer (Fig. 2 *A*, *Left* panel), in line with the pattern of insertion proposed above. Interestingly, however, when this experiment was repeated in the Δ*slaA* mutant, the newly expressed proteins formed islands that only partially covered the cell surface (Fig. 2 *A*, *Right* panel). We hypothesized that this partial S-layer coverage phenotype observed in the Δ*slaA* SlaA-HA rescue strain might reflect the ability of S-layer proteins to spontaneously assemble into a lattice. This ability to self-assemble would enable newly exported SlaA-HA protein to randomly fill in gaps in the lattice that form during growth or as the result of mechanical disruption to maintain even and complete coverage of the membrane by the S-layer (Fig. 2 *B*, *Left* panel). In the Δ*slaA* SlaA-HA strain, by contrast, SlaA-HA was lost from the cell unless it was able to associate with an existing S-layer lattice. As a consequence, patches of SlaA-HA expanded outward through self-assembly until they coated the entire cell surface (Fig. 2 *B*, *Right* panel).

To better probe this process of self-assembly, we followed the induction of SlaA-HA in Δ*slaA* mutant cells over an 8 h time-course. Over this period, there was a visible increase in the coverage of the cell surface with SlaA-HA (Fig. 2 *C* and *D*). To verify that this mode of assembly was not specific to the Δ*slaA* mutant, MW001 cells were treated with Proteinase K to remove the endogenous S-layer, prior to the induction of SlaA-HA expression (Fig. 2*E*). Again, the full S-layer coat was restored via nucleation and lattice growth in support of this model of SlaA assembly (Fig. 2*B*).

These experiments using HA-tagged SlaA protein assayed the ability of newly expressed protein to be inserted into the S-layer following its export across the membrane via the translocon. To determine whether prefolded SlaA protein could also be incorporated into a growing S-layer lattice, we attempted to coat cells with exogenous labeled SlaA. For this experiment, S-layer proteins were extracted from wild-type *S. acidocaldarius* cells using a slightly modified version of previously published protocols (*SI Appendix*, Fig. S2*E*; see also *Materials and Methods*) (24, 51). The purified SlaA, which has been shown to exist in solution in part as reassembly-competent dimers (23–25), were then covalently conjugated with a fluorescent NHS ester dye and incubated with S-layer mutant strains. As assayed by flow cytometry, this method efficiently labeled the surface of Δ*slaA* mutant cells, where the membrane anchor SlaB is likely free to capture exogenous soluble SlaA, but not in Δ*slaB* mutants or in control cells that already possess a complete SlaA coat (*SI Appendix*, Fig. S2*F*). When visualized using light microscopy, fluorescently-conjugated exogenous SlaA was seen forming islands on the surface of Δ*slaA* mutant cells but was not seen associating with the SlaA-HA overexpression strain, which likely lacks free surface SlaB (Fig. 2*F*). Strikingly, when exogenous labeled protein was added to Δ*slaA* SlaA-HA strains expressing relatively low levels of HA-tagged SlaA, the labeled SlaA was recruited to the edges of the SlaA-HA patches as expected if the endogenously forming lattice is able to extend at its margins via the gradual recruitment of assembly-competent prefolded SlaA proteins. Taken together, these data demonstrate that a complete S-layer in *S. acidocaldarius* is likely generated and maintained during cell growth via the energetically favorable process of self-assembly in a manner that does not depend on local SlaA translation and export.

### The S-Layer Influences Cell Division in *S. acidocaldarius*.

**Fig. 3.**
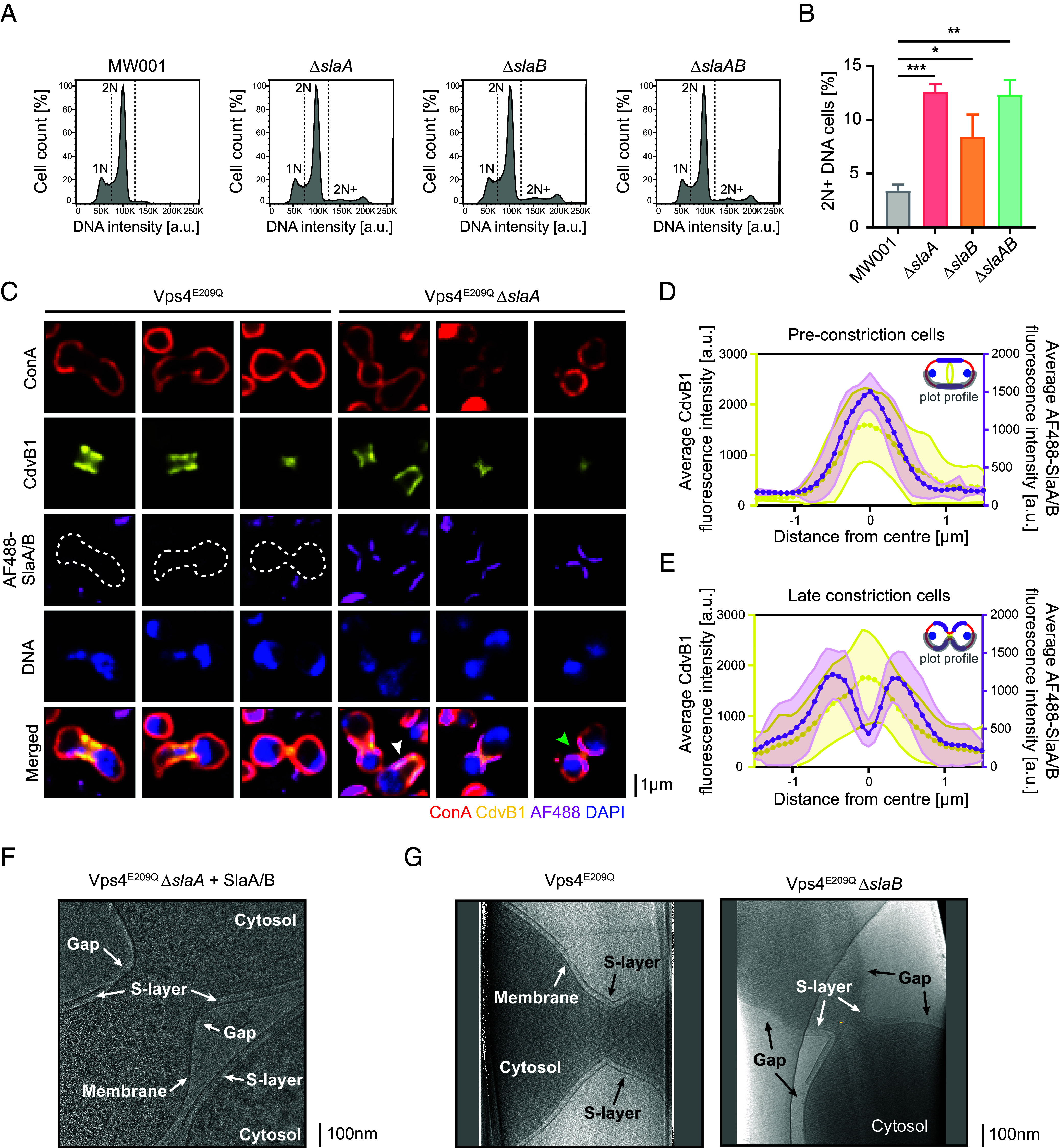
The S-layer is implicated in cell division and localizes preferentially to the division bridge. (*A*) Representative flow cytometry histograms of DNA content in asynchronous cultures of MW001 and S-layer mutants. (*B*) Quantification of percentage of cells exhibiting more than 2 N DNA content in the flow cytometry experiments (N = 3, n = 5.0 × 10^5^ events each). Shown here are means ± SD (MW001: 3.4 ± 0.50%; Δ*slaA*: 12.6 ± 0.60%; Δ*slaB*: 8.4 ± 1.7%; Δ*slaAB*: 12.3 ± 1.1%). *P*-values were derived using Welch’s *t* test (N = 3; *P ≤ 0.05; **P ≤ 0.01; ***P ≤ 0.001). (*C*) Representative immunofluorescence images of MW001 and the Δ*slaA* mutant overexpressing the dominant negative Walker B mutant Vps4^E209Q^. Fluorescently-labeled exogenous S-layer proteins were added to identify gaps in the S-layer of these cells. Shown here are representative single-plane spinning-disk confocal images. Plot profiles of the average fluorescence intensities of CdvB1 (yellow) and fluorescently-labeled exogenous S-layer proteins (magenta) in preconstriction cells (*D*) or late constriction cells (*E*). A diagram showing the plot profile used indicated in gray is shown. Shown here are means, and the SD are indicated by the shaded areas (n = 25 cells each). Examples of preconstriction and late constriction cells are indicated by white and green arrowheads respectively in (*C*). (*F*) Representative cryo-EM image of Vps4^E209Q^ Δ*slaA* mutants incubated with unlabeled exogenous S-layer proteins. See also *SI Appendix*, Fig. S3*G*. (*G*) Representative cryo-ET slices of MW001 and the Δ*slaB* mutant overexpressing the Walker B dominant negative mutant Vps4^E209Q^ showing the S-layer localization on the cell surface. See also *SI Appendix*, Fig. S3*H* and Movies S1–S4.

Previous studies in related Sulfolobales have implicated the S-layer in cell division (39, 40). To test whether or not SlaA plays an analogous role in *S. acidocaldarius*, we performed a flow cytometry analysis to assess the DNA content of our panel of S-layer deletion mutants during exponential growth. Populations of exponentially growing cells in all conditions have a relatively similar 1 N and 2 N DNA content, corresponding to the G1 and G2 phases of the cell cycle (Fig. 3*A*). In populations of cells lacking either SlaA and/or SlaB, however, we observed a significant, if slight, increase in the proportion of cells containing an abnormal >2 N DNA content (Fig. 3 *A* and *B*)—a phenotype indicative of a mild division defect.

These observations led us to look more closely at the role of the S-layer in the shape changes that accompany cytokinesis. To image division bridges in detail, we arrested control and Δ*sla* mutant cells at different stages of division using a dominant negative Vps4 mutant with an E209Q point mutation in the Walker B motif (Vps4^E209Q^) that blocks ATP hydrolysis and ESCRT-III ring remodeling (4) (*SI Appendix*, Fig. S3*A*). Exogenous labeled SlaA was then added to these cells to probe for gaps in the S-layer (*SI Appendix*, Fig. S3*B*). In Vps4^E209Q^ control cells that express endogenous SlaA, the cells were not efficiently labeled by the addition of exogenous labeled S-layer proteins, implying a paucity of gaps in the lattice that can be filled by exogenous protein, even at sites close to the division bridge where the lattice is deformed by ESCRT-III-dependent medial constriction (Fig. 3 *C*, *Left* panels). By contrast, exogenously-labeled S-layer proteins formed extended lattices on Vps4^E209Q^ Δ*slaA* cells. In these instances, labeled S-layer proteins preferentially coated division bridges (Fig. 3 *C*, *Right* panels) (95.0 ± 0.82% of dividing cells with AF488 signal at the bridge; N = 3, n = 100 cells). To test whether this local accumulation of labeled protein at cell–cell bridges might reflect the S-layer lattice’s preference for membranes with a defined curvature or specific regions of the cell surface, we took advantage of the fact that Vps4^E209Q^ Δ*slaA* cells assume a variety of shapes to compare the labeling of cells in the preconstriction stage where they take up an extended oval shape, to dumbbell-shaped cells in late constriction stage.

When we examined S-layer lattices coating Vps4^E209Q^ Δ*slaA* cells arrested prior to furrow formation, the bias was striking. Plot profiles of the cell surface show that S-layer lattices in these cells span the entire midzone of the cell, marked by the ESCRT-III homolog CdvB1 ring, but are absent from cell tips (Fig. 3*D* and *SI Appendix*, Fig. S3*C*). This distribution was confirmed by a correlation analysis, which revealed a preference of the exogenous labeled S-layer for regions of lower curvature—something surprising given the overall positive curvature of *Sulfolobus* cells (*SI Appendix*, Fig. S3*D*). Next, we looked at the localization of exogenous SlaA in dumbbell-shaped Vps4^E209Q^ Δ*slaA* cells. In this case, exogenous S-layer accumulated at the division bridge, but tended to be excluded from the very center of the bridge, where the local curvature is highest (Fig. 3*E* and *SI Appendix*, Fig. S3*E*). Taken together, these results suggest that, while the S-layer is flexible enough to coat the entire surface of cells as they grow and divide, it may have a preference for regions of the cell surface that have lower curvature.

To test whether this pattern of exogenous SlaA in dividing cells follows the distribution of SlaB, we expressed HA-SlaB in the MW001 and Δ*slaA* background strains (*SI Appendix*, Fig. S3*F*). When probed using an anti-HA antibody, SlaB was detected across the cell surface, from the cell tips to the midzone, making it unlikely that the preferential localization of exogenous SlaA to the division bridge reflects limits in the local availability of free SlaB.

A cryo-EM analysis confirmed that the exogenous-added labeled SlaA was able to form a proper S-layer lattice on the surface of Δ*slaA* (Fig. 3*F*), similar to that seen in the control (Fig. 1*C*). In addition, this analysis revealed that, while soluble S-layer proteins are able to form relatively flat lattices on the surface of Vps4^E209Q^ Δ*slaA* cells, the S-layer lattice tends to be excluded or is less uniform in regions of the division bridge exhibiting very high local curvature (Fig. 3*F* and *SI Appendix*, Fig. S3*G*).

To test whether or not endogenously expressed S-layer proteins have a similar preference for division bridges, we looked at the surface patches of SlaA remaining in Δ*slaB* cells (Fig. 1 *C*, *Third* panel). Again, when these cells were arrested at division by expression of Vps4^E209Q^, we observed a similar bias by cryo-ET (Fig. 3*G* and *SI Appendix*, Fig. S3*H*; see also Movies S1–S4). While a normal intact S-layer was observed enveloping the entire surface of control Vps4^E209Q^ cells, in Vps4^E209Q^ Δ*slaB* cells the patches of endogenous SlaA S-layer were preferentially localized close to the division bridge (71.43% of the S-layer was at the division bridge; 15/21 cells). In contrast to exogenous labeled S-layer, the S-layer formed in Vps4^E209Q^ Δ*slaB* cells tended to remain continuous across the middle of constricted bridges, and was only excluded from the central region in a relatively small fraction of cases (23.81%—5/21 cells; 1/21 had no S-layer).

Taken together these data show that, while the S-layer remains intact at the division site of wild-type cells, with few gaps, in cells with extended division bridges, a partial S-layer preferentially coats the membrane at the division bridge and, when exogenously added, preferentially binds to these regions of lower curvature.

### Cell Division in S-Layer Mutants.

Having shown that the S-layer has a preference for the regions of the membrane on either side of the midzone of dividing cells, it was important to test whether the S-layer alters the division machinery bound to the cytoplasmic side of these division bridges. The loss of the S-layer in Δ*slaA*, Δ*slaB,* and Δ*slaAB* mutants did not visibly alter the localization of the division machinery itself, nor the stepwise changes in the composition of the ESCRT-III rings (CdvB, CdvB1, and CdvB2) driving division (5, 52–55), when assayed using either via flow cytometry (*SI Appendix*, Fig. S4*A*) or immunofluorescence (*SI Appendix*, Fig. S4*B*).

**Fig. 4.**
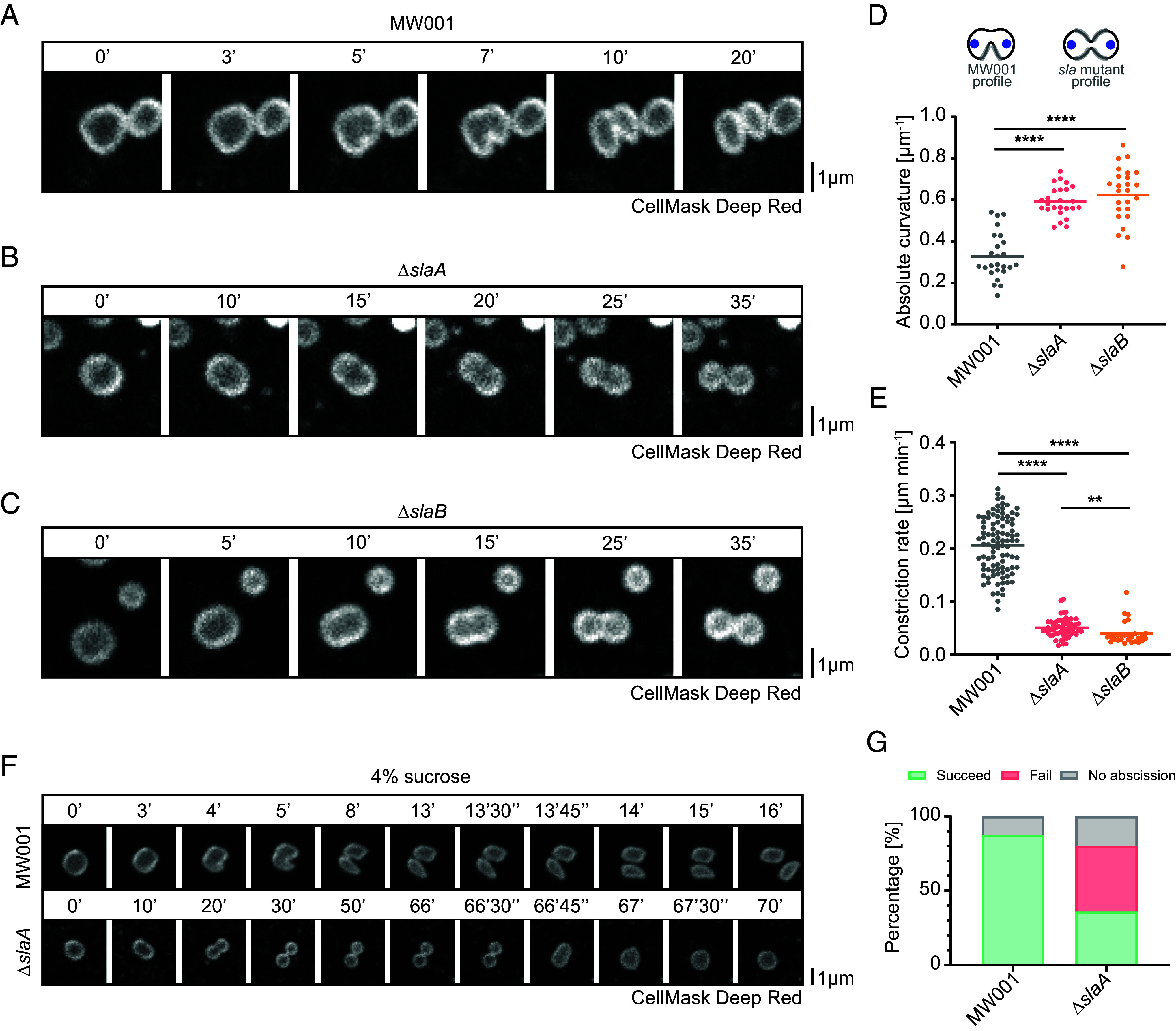
S-layer at the division bridge ensures rapid constriction and abscission during cell division. Time-lapse imaging of (*A*) the uracil auxotrophic control strain MW001, (*B*) Δ*slaA* mutant and (*C*) Δ*slaB* mutant undergoing cell division. Representative division montages are shown. Cells were stained with CellMask Deep Red Plasma Membrane stain and imaged at 75 °C in Brock medium supplemented with uracil. (*D*) Scatter plot with means indicated showing the quantification of the average curvature of the cell surface adjacent to the constricting division bridge in MW001 and the S-layer mutants imaged under live conditions (MW001: 0.326 ± 0.109 μm^−1^; Δ*slaA*: 0.591 ± 0.0710 μm^−1^; Δ*slaB*: 0.624 ± 0.134 μm^−1^). Diagrams showing the profile used for measurements are shown above. *P*-values were derived using Welch’s *t* test (n = 25 cells each; ****P ≤ 0.0001; *n.s.*, no significance). (*E*) Scatter plot with means indicated showing the rate of constriction of MW001 and S-layer mutants (MW001: 0.2058 ± 0.0527 μm min^−1^; Δ*slaA*: 0.0507 ± 0.0177 μm min^−1^; Δ*slaB*: 0.0398 ± 0.0210 μm min^−1^). *P*-values were derived using Welch’s *t* test (n ≥28 cells each; ****P ≤ 0.0001; **P ≤ 0.01). (*F*) Time-lapse imaging of MW001 (*Top*) and Δ*slaA* mutant (*Bottom*) performed at 75 °C in Brock medium supplemented with uracil, in the presence of 4% sucrose. Cells were stained with CellMask Deep Red Plasma Membrane stain. Representative montages are shown. (*G*) Quantification of cell division failure in MW001 control cells and Δ*slaA* mutant dividing in 4% sucrose medium imaged under live conditions (n ≥ 25; MW001: 87.5% succeed, 12.5% no abscission; Δ*slaA*: 36% succeed, 44% fail, 20% no abscission).

Next, as a more direct test of the role of the S-layer in cytokinesis, we imaged control MW001 cells and Δ*sla* mutant cells labeled with CellMask Plasma Membrane stain as they grew and divided. Under these conditions, control MW001 cells had an irregular roughly spherical shape during interphase, but then rapidly transitioned into an angular shape with a flat division furrow during cytokinesis (Fig. 4*A*). This was due to the fact that, as previously reported (37), in control MW001 cells the furrow deepens at a relatively constant rate, via a process that resembles slicing, to produce two daughter cells of similar sizes within a period of approximately 10 min (Fig. 4*A*) (4, 56). The contrast between division in MW001 cells and division in cells lacking SlaA or SlaB was striking. Δ*sla* mutant cells were nearly spherical in interphase and remained round or dumbbell shaped throughout the process of cell division (Fig. 4 *B* and *C*). This difference was significant as shown by the quantification of local curvature in regions of the cell close to the division bridge (Fig. 4*D*). Moreover, at intermediate timepoints, cells exhibited smoothly curved division bridges and daughter cells were significantly rounder in Δ*sla* mutant cells than they were in the corresponding control cells (*SI Appendix*, Fig. S4*C*).

To assay the role of the S-layer in furrow progression, we measured the time taken from the beginning until the end of cytokinesis. This revealed a dramatic and significant reduction in the constriction rate of the furrow in the absence of a complete S-layer (Fig. 4*E*). This was accompanied by an increase in the rates of division error—such as markedly asymmetric divisions (Fig. 3*A*). Finally, to assess whether this mechanical role of the S-layer might be more important under more physically challenging conditions, we subjected control and Δ*sla* mutant cells to osmotic shock. While division failures were rare in control cells shifted to hyperosmotic media containing 4% sucrose (twenty times the standard culture medium concentration), a significant number of division bridges collapsed at late stages just prior to abscission in cells lacking an S-layer (Fig. 4 *F* and *G*).

## Discussion

The self-assembly of large-scale protein structures has been studied in a host of organisms including the bacteria flagella (57), virus capsids (58), or even prions and amyloid fibrils in higher eukaryotes (59, 60). Such self-assembling one- or two- dimensional lattices play a particularly important role in membrane remodeling in eukaryotic membrane trafficking, where the self-assembly of clathrin, COPI, and COPII proteins allows coating of membranes, acting as a first step in vesicle formation (61–63). In these cases, the regulated formation of a passive, self-assembling protein coat [e.g. Sec23/24, Sec13/31 (64), or clathrin (65)] generates positive membrane curvature. Energy-dependent processes (driven by the Sar1 GTPase, the Dynamin GTPase, or actin filament formation), then catalyze membrane scission (66, 67). These examples show how self-assembling polymers on the surface of a membrane can collaborate with cytoplasmic force-generating systems to induce membrane deformation and scission.

In a similar vein, here we show how the energy-independent self-assembly of an SlaA lattice on membrane anchors on the surface of *Sulfolobus* cells assists in the cell deformation that accompanies cytokinesis. In the case of *Sulfolobus*, AAA-ATPase Vps4-dependent remodeling of composite ESCRT-III polymers acts together with the overlying S-layer to drive cytokinesis and abscission, leading to the formation of two daughter cells. Interestingly, the S-layer lattice that performs this role in division is assembled as a coat on interphase cells that are near spherical. Since partial SlaA lattices seem to prefer to bind to regions of the membrane that have a lower curvature, this suggests the possibility that there is stored elastic energy in the positively curved interphase lattice that is relieved during cytokinesis to help to form the furrow as a prelude to scission. This resembles what has been proposed to happen in eukaryotic cells when the mechanical energy stored in flat clathrin lattices on the plasma membrane assists in the formation of endosomes (68, 69), and in bacterial cells where the build up of stress in the cell-wall aids the mechanical splitting apart of daughter cells during cytokinesis (70). Despite having a preference for sites of lower curvature, the *Sulfolobus* S-layer must be flexible, since in wild-type cells it completely covers the entire cell surface at all times—and completely coats small extracellular vesicles generated by *Sulfolobus* cells at division (37).

Importantly, we found no evidence of cell division being aided by new local S-layer insertion at the site of membrane furrowing in dividing *Sulfolobus* cells (*SI Appendix*, Fig. S2*D*). Instead, the S-layer appears to form a packed lattice that is maintained by local SlaA insertion into gaps as cells grow, and which can change its conformation to accommodate changes in membrane shape to completely coat the division bridge—as shown using cryo-EM (Fig. 3*G* and *SI Appendix*, Fig. S3*H*). Thus, the way the S-layer in *Sulfolobus* aids division appears to be a mechanical feature of the fully assembled lattice, not the result of dynamic local insertion—as it is in the case of Euryarchaea like *Haloferax* (17, 32), where the S-layer is laid down at the division plane to assist in division. In line with this, we did not observe any effects of S-layer proteins on the localization or dynamics of the cytoplasmic ESCRT-III division ring (*SI Appendix*, Fig. S4 *A* and *B*). Nevertheless, we note that there was a clear correlation between the pattern of CdvB1 recruitment to the internal side of the membrane in dividing Vps4^E209Q^ Δ*slaA* cells and the pattern of exogenously added SlaA (Fig. 3 *C*–*E*); suggesting the possibility that SlaA lattices prefer membranes that are supported by an underlying ESCRT-III coat.

Taking together, these data lead us to propose that the S-layer flattens the membrane at the furrow on either side of the division bridge of dividing cells, where CdvB1 accumulates, to accelerate furrow ingression and scission (Fig. 4 *D* and *E*). This fits with the observed preference of SlaA lattices for planar packing in vitro (23, 24). It also explains why, in the absence of an S-layer, cells appear to divide by medial ring constriction, whereas control cells divide in a process that looks like slicing (Fig. 4 *A*–*C*). The idea that the S-layer plays a simple mechanical role in cytokinesis is supported by its ability to protect cells subjected to mechanical shocks induced by changes in osmotic pressure from division failure (Fig. 4 *F* and *G*). Although much harder to standardize, similar results were obtained when MW001 and Δ*sla* mutant cells were subjected to mild mechanical compression.

In summary, our results show that S-layer monomers can self-assemble on the cell surface. This fits with studies that have demonstrated the ability of S-layer proteins to self-assemble in vitro, either in solution or on artificial substrates (71–75). In cells, this process of self-assembly generates a relatively rigid lattice that expands as cells grow. Most strikingly of all, this structure both protects the bounding membrane of cells from environmental hazards, while also aiding their rapid and efficient division—two properties that one might have expected to be at odds with one another.

## Materials and Methods

### Strains, Culture Media, and Growth Conditions.

All *S. acidocaldarius* strains were grown at 75 °C, pH 3.0, in Brock medium supplemented with 0.1% (w/v) NZ-amine and 0.2% (w/v) sucrose (76). Strains used in this study are found in *SI Appendix*, Table S1. The uracil auxotroph strain MW001 and all deletion mutants were further supplemented with 4 μg/mL uracil (38). Solid Brock medium was prepared by mixing preheated twice concentrated liquid Brock medium pH 5.0 supplemented with 0.2% NZ-amine (w/v), 0.4% sucrose (w/v), 20 mM CaCl_2_, and 1.2% Gelrite (w/v), in a 1:1 ratio.

All liquid cultures were passaged up to 2 times and grown to exponential phase to an OD_595_ of 0.10 to 0.25 prior to use in all experiments, unless otherwise stated. Cells were fixed by three stepwise additions of 4 °C ethanol to a final concentration of 70% and then stored at 4 °C, as described previously (77). Induction of protein expression in overexpression strains and rescue mutants was performed by addition of L-arabinose to the culture medium to a final concentration of 0.2% (w/v) in exponentially growing cultures for 4 h.

### Molecular Genetics.

Deletion mutants were constructed as described previously, using the pSVA406 vector system (38). Transformation of electrocompetent *S. acidocaldarius* was performed following established protocols for *Sulfolobus* genetics (38). Further details on molecular genetics, generation of electrocompetent cells and whole genome sequencing can be found in the *SI Appendix*, *Extended Materials and Methods*.

The SlaAB double deletion mutants was constructed by deletion of the entire operon from the start codon of SlaA (*saci2355*) to the stop codon of SlaB (*saci2354*) inclusive in electrocompetent MW001, using the protocol described above. The SlaB Saci1846 double deletion mutant was constructed by deletion of SlaB followed by a subsequent deletion of *saci1846* in electrocompetent Δ*slaB* cells.

Overexpression and rescue mutant constructs were cloned into the pSVAaraHA-stop plasmid backbone, typically between the NcoI and XhoI restriction sites (78). The SlaA overexpression construct was created using two fragments, with a silent A-G point mutation introduced at DNA position 2079 of the SlaA open reading frame via primer design to create an internal BamHI site for cloning between NcoI and BamHI, followed by BamHI and XhoI for the second fragment. This was done to avoid the endogenous NcoI site in the gene that is required for restriction cloning into the pSVAaraHA-stop plasmid. The HA-SlaB construct was procured commercially as a double-stranded DNA fragment (gBlock, Integrated DNA Technologies) flanked by NcoI and XhoI restriction sites, with the HA tag (YPYDVPDYA) inserted after the signal sequence (amino acid residue S24) of SlaB. Vps4^E209Q^ dominant negative mutant overexpression construct was obtained from the lab collection (4).

### Cryo-EM and cryo-ET Sample Preparation, Data Collection, and Image Analysis.

Samples for cryo-EM and cryo-ET were prepared and performed as described previously (17, 79, 80), using a Vitrobot Mark IV (Thermo Fisher Scientific). Cryo-EM images were collected using a Glacios TEM (Thermo Fisher Scientific) operating at 200 kV using a Falcon 3 (Thermo Fisher Scientific) direct electron detector. All electron cryomicrographs shown have been lowpass filtered to improve visibility of the cell envelope and S-layer.

Tilt-series data were acquired using a Krios G3 TEM (Thermo Fisher Scientific) operating at 300 kV using a K3 (Gatan) direct electron detector equipped with a Quantum energy filter (Gatan) with a slit width of 20 eV. Further details on cryo-EM and cryo-ET protocols can be found in the *SI Appendix*, *Extended Materials and Methods*.

### Immunofluorescence Labeling.

Ethanol fixed cells were washed with phosphate-buffered saline supplemented with 0.2% Tween-20 (Sigma-Aldrich, P9416) and 3% Bovine Serum Albumin (Sigma-Aldrich, A7030) (PBSTA) for rehydration and blocking. Cells were probed with primary and secondary antibodies (*SI Appendix*, Table S2) in PBSTA. Labeling of cell surface was performed by incubating with 5 μg/mL concanavalin A conjugated to Alexa Fluor 647 (Thermo Fisher Scientific, C21421) during secondary antibody incubation. Stained cells were resuspended in 200 μL of PBST containing either 1 μg/mL DAPI (Thermo Fisher Scientific, 62248) for imaging, or 2 μM Hoechst (Thermo Fisher Scientific, 62249) for flow cytometry experiments. Full details on immunofluorescence labeling can be found in the *SI Appendix*, *Extended Materials and Methods*.

### Microscopy.

Fixed cells were imaged in Lab-Tek chamber slides (Thermo Fisher Scientific, 177437PK) coated with 2% polyethyleneimine for 2 h at 37 °C. Chambers were rinsed with Milli-Q water twice before addition of 200 μL of stained cells. Cells were immobilized by centrifugation for 1 h at 750 rcf by taping the chamber slides down to a centrifuge microplate adaptor. Imaging was performed using a Nikon Eclipse Ti2 inverted microscope equipped with a Yokogawa SoRa scanner unit and Prime 95B scientific complementary metal-oxide semiconductor (sCMOS) camera (Photometrics). All images were acquired using a 100x oil immersion objective (Apo TIRF 100×/1.49, Nikon) with immersion oil (Nikon, Type F2). A further 2.8x magnification was achieved using the 2.8x lens in the SoRa unit. Z-steps were set at 0.22 μm. All analysis and z-axis projections were performed in ImageJ. Quantification of ConA intensity was performed in ImageJ using the segmented line function with a line width of 2 pixels.

### Flow Cytometry.

Fixed cells were stained as described above and DNA labeled with 2 μM Hoechst. Flow cytometry experiments were performed on a BD Biosciences LSRFortessa. Laser excitation wavelengths of 355, 488, 561, and 633 nm were used in conjunction with the emission filters 450/50, 530/30, 586/15, and 670/14, respectively. Both side and forward scatter information was recorded as well. All analysis of flow cytometry data was performed on FlowJo v10 software.

### Labeling of Cell Surface with NHS Ester Dyes.

*S. acidocaldarius* cells were washed and resuspended in 50 mM HEPES pH 8.0 buffer. Pulse labeling was performed by addition of Alexa Fluor 488 NHS succinimidyl ester dye (Invitrogen, A20000). Next, the cells were washed and diluted into prewarmed Brock medium, and allowed to grow for 2 h. The process was then repeated with Alexa Fluor 647 NHS succinimidyl ester dye (Invitrogen, A20009) for chase labeling, and cells were fixed in ethanol as described previously. Full details on the labeling protocol and optimization can be found in the *SI Appendix*, *Extended Materials and Methods*.

Quantification of pulse and chase labeling, as well as labeling optimization, was performed in ImageJ using the segmented line function with a line width of 2 pixels. Line plots were generated in ImageJ and the raw values exported for processing in Microsoft Excel and Graphpad Prism 7 software.

### Proteinase K Treatment.

30 mL of exponentially growing SlaA-HA overexpression strains were induced with arabinose for 4 h. Cells were then pelleted via centrifugation at 4,000 rcf for 5 min. Cell pellets were transferred to a clean microcentrifuge tube and washed twice in 50 mM HEPES buffer pH 8.0. The pellet was then resuspended in 1 mL of 50 mM HEPES buffer pH 8.0, and 20 μL of 20 mg/mL proteinase K added. This was incubated in an Eppendorf thermomixer at 60 °C for 30 min with 500 rpm shaking. Next, proteinase K was removed by washing the cells once in 1 mL of 50 mM HEPES buffer pH 8.0 and once in 1 mL of prewarmed Brock medium. This was then diluted into 25 mL of prewarmed Brock medium and allowed to grow for 4 h. The cells were next fixed in ethanol as described previously.

### S-Layer Purification.

S-layer purification was performed as described previously (24, 51), with minor modifications for compatibility with benchtop centrifugation. Cell pellets were resuspended in 10 mM HEPES buffer pH 7.0 containing 2 mM EDTA and 1 mM PMSF (HEPES wash buffer), supplemented with 0.15% SDS. 100 μg/μL DNaseI (ITW reagents, A3778.0100) and 4 mM MgCl_2_ was added and the mixture incubated for 1 h at 37 °C. Next, 10% SDS solution was added to bring the final SDS concentration to 2% and the mixture was left overnight on a roller mixer at room temperature.

The mixture was next centrifuged for 1 h at room temperature at 4,000 rcf and the supernatant discarded. The pellet was washed with HEPES wash buffer +2% SDS for 1 h at 75 °C with 200 rpm shaking in the incubator, followed by centrifugation for 1 h at room temperature at 4,000 rcf and the supernatant discarded. This wash was repeated for a further two times, before a final resuspension in HEPES wash buffer +2% SDS overnight. The following day, the pellet was then washed six times in milli-Q water before the pellet was solubilized in 20 mM Na-carbonate buffer pH 10 at 75 °C for 30 min. Detailed protocols for the S-layer purification can be found in the *SI Appendix*, *Extended Materials and Methods*.

### S-layer In Vivo Assembly.

10 to 30 μg of purified S-layer proteins were diluted 1:1(v/v) with 50 mM HEPES pH 7.0. Next, 1 μL of a 1 mg/mL stock of Alexa Fluor 488 NHS succinimidyl ester dye (Invitrogen, A20000) in DMSO was added and the mixture incubated for 15 min at room temperature with gentle agitation. Then, 3 mL of cells in the exponential growth phase was transferred to a 15 mL falcon tube, and labeled S-layer proteins were added. This was incubated at 75 °C with shaking for 1 h. The cells were next fixed in ethanol as described previously. For cryo-EM analysis of S-layer in vivo assembly, unlabeled S-layer proteins were added instead.

### ConA Pull-Down and Western Blotting.

MW001, SlaA-HA, and HA-SlaB cell pellets were resuspended in IP lysis buffer (25 mM HEPES pH 7.5, 10 mM KCL, 1 mM CaCl_2_, 1 mM MnCl_2_) supplemented with 1× RIPA Lysis buffer (Merck, 20 to 188) and 1× cOmplete protease inhibitor (Roche, 11836170001). The cells were next lysed in a Bioruptor Plus sonication device at 4 °C. Lysed cells were then centrifuged for 5 min at 10,000 rcf at 4 °C to pellet cellular debris.

Preequilibrated concanavalin A beads (Antibodies-online, ABIN6952467) were added to each prepared supernatant and ConA pull-down performed on a rotating mixer at 4 °C. Following that, the beads washed with ice-cold IP lysis buffer before being resuspended in 1x NuPAGE LDS sample buffer (Thermo Fisher Scientific, NP0007) containing 10% β-mercaptoethanol. Cell lysate control and ConA pull-down samples were incubated at 98 °C for 5 min before loaded and ran on a NuPAGE 4 to 12% Bis-Tris gels (Invitrogen). Proteins were transferred to nitrocellulose membranes and blocked using 5% milk in PBST. Incubation with primary and IRDye secondary antibodies (*SI Appendix*, Table S2) was performed in PBST +5% milk. The membrane was imaged using a Bio-Rad ChemiDoc system. Detailed protocols for pull-down and Western Blotting can be found in the *SI Appendix*, *Extended Materials and Methods*.

### Mass Spectrometry.

ConA pull-down of *S. acidocaldarius* wild-type DSM639 whole cell lysate was performed using the protocol described above. ConA beads were resuspended in AMBIC 0.1% RapiGest and digested for protein identification by mass spectrometry. Cysteines were reduced by addition of DTT, followed by alkylation and trypsin in-bead digestion. The following day, trifluoracetic acid was added to stop digestion and induce RapiGest degradation. The mixture was centrifuged and the supernatant used for mass spectrometry analysis.

LC–MS/MS was performed on an Ultimate U3000 HPLC (ThermoFisher Scientific) hyphenated to an Orbitrap QExactive Classic mass spectrometer (ThermoFisher Scientific). Peptides were trapped on a C18 Acclaim PepMap 100 (5 µm, 300 µm × 5 mm) trap column (ThermoFisher Scientific) and eluted onto an IonOpticks Aurora Ultimate column (1.7 µm, 75 µm × 250 mm) using 52 min gradient of acetonitrile (7 to 40%). Full protocols used for mass spectrometry can be found in the *SI Appendix*, *Extended Materials and Methods*.

### Trichloroacetic Acid Precipitation of SlaA from Growth Medium.

10% (v/v) trichloroacetic acid (Sigma-Aldrich, T6399) was added to the filtered growth medium and the mixture incubated on ice for 1 h with intermittent mixing. Precipitated proteins were pelleted via centrifugation, washed in ice-cold acetone, and pelleted again. The pellet was dried in a Eppendorf Vacufuge plus vacuum concentrator. Precipitated proteins were then resuspended in 1x NuPAGE LDS sample buffer (Thermo Fisher Scientific, NP0007) containing 10% β-mercaptoethanol, boiled, and ran on an SDS-PAGE gel as described previously. Proteins were visualized via staining of the gel with InstantBlue Coomassie Protein Stain (Abcam, ab119211). Detailed protocols for TCA precipitation of soluble proteins from growth medium can be found in the *SI Appendix*, *Extended Materials and Methods*.

### Live Cell Microscopy.

Live cell imaging was performed using the Sulfoscope set-up as described previously (4, 37, 77) on Attofluor chambers (Invitrogen, A7816) assembled with 25 mm coverslips. Images were acquired with a 60× oil immersion objective (Plan Apo 60×/1.45, Nikon) using a custom formulated immersion oil for high-temperature imaging (maximum refractive index matching at 70 °C, n = 1.515 ± 0.0005; Cargille Laboratories). Images were acquired at intervals of 15 s for 2.5 h or until any cell death was observed. XY drift was corrected after acquisition using the ImageJ plugin StackReg (81). Full details of live cell microscopy set-up and protocols can be found in the *SI Appendix*, *Extended Materials and Methods*.

### Curvature and Roundness Measurements.

Curvature measurements were performed using the Kappa plugin on Fiji (82). Data points were exported and analyzed on Microsoft Excel and GraphPad Prism10. Spearman’s rank correlation coefficient was calculated between the absolute curvature and exogenous S-layer labeling fluorescence intensity to determine the relationship between the two variables. Quantification in live cells was obtained by averaging the curvatures on the regions adjacent to the division bridge in late constriction cells. Daughter cell shape was analyzed in Fiji following thresholding and creation of a binary mask. Cell roundness is reported as 4 * Area/(π * MajorAxis^2^).

### Statistical Analysis.

All statistical analysis was performed on Microsoft Excel or GraphPad Prism 10 software. All data presented are shown as mean ± SD, unless otherwise specified. Significance was defined as *P* ≤ 0.05. Significance levels used were *P ≤ 0.05, **P ≤ 0.01, ***P ≤ 0.001 and ****P ≤ 0.0001.

## Supplementary Material

Appendix 01 (PDF)

Movie S1. **Cryo-ET of MW001 expressing Vps4_E209Q_**. The S-layer is present with no visible gaps on the cell surface of MW001 expressing the Walker B dominant negative mutant Vps4_E209Q_ arrested at division. Scale bar represents 100nm.

Movie S2. **Cryo-ET of MW001 expressing Vps4_E209Q_**. The S-layer is present with no visible gaps on the cell surface of MW001 expressing the Walker B dominant negative mutant Vps4_E209Q_ arrested at division. Scale bar represents 100nm.

Movie S3. **Cryo-ET of *ΔslaB* mutants expressing Vps4_E209Q_**. The S-layer patches present in *ΔslaB* mutants preferentially localizes to the division bridge in *ΔslaB* cells expressing the Walker B dominant negative mutant Vps4_E209Q_ arrested at division. Scale bar represents 100nm.

Movie S4. **Cryo-ET of MW001 expressing Vps4_E209Q_**. The S-layer patches present in *ΔslaB* mutants preferentially localizes to the division bridge in *ΔslaB* cells expressing the Walker B dominant negative mutant Vps4_E209Q_ arrested at division. Scale bar represents 100nm.

## Data Availability

All study data are included in the article and/or supporting information.

## References

[1] B. Baum, A. Spang, On the origin of the nucleus: A hypothesis. Microbiol. Mol. Biol. Rev. **87**, e00186-21 (2023). 38018971 10.1128/mmbr.00186-21 PMC10732040

[2] L. Eme, A. Spang, J. Lombard, C. W. Stairs, T. J. G. Ettema, Archaea and the origin of eukaryotes. Nat. Rev. Microbiol. **15**, 711–723 (2017). 29123225 10.1038/nrmicro.2017.133

[3] A. Cezanne, S. Foo, Y.-W. Kuo, B. Baum, The archaeal cell cycle. Annu. Rev. Cell Dev. Biol. **10**, 1–23 (2024), 10.1146/annurev-cellbio-111822-120242. PMC7617429 38748857

[4] F. Hurtig , The patterned assembly and stepwise Vps4-mediated disassembly of composite ESCRT-III polymers drives archaeal cell division. Sci. Adv. **9**, eade5224 (2023). 36921039 10.1126/sciadv.ade5224 PMC10017037

[5] G. Tarrason Risa , The proteasome controls ESCRT-III-mediated cell division in an archaeon. Science **369**, eaaz2532 (2020). 32764038 10.1126/science.aaz2532 PMC7116001

[6] T. A. M. Bharat, A. von Kügelgen, V. Alva, Molecular logic of prokaryotic surface layer structures. Trends Microbiol. **29**, 405–415 (2021). 33121898 10.1016/j.tim.2020.09.009 PMC8559796

[7] U. B. Sleytr, B. Schuster, E.-M. Egelseer, D. Pum, S-layers: Principles and applications. FEMS Microbiol. Rev. **38**, 823–864 (2014). 24483139 10.1111/1574-6976.12063 PMC4232325

[8] S.-V. Albers, B. H. Meyer, The archaeal cell envelope. Nat. Rev. Microbiol. **9**, 414–426 (2011). 21572458 10.1038/nrmicro2576

[9] E. Johnston, B. Isbilir, V. Alva, T. A. M. Bharat, J. P. K. Doye, Punctuated and continuous structural diversity of S-layers across the prokaryotic tree of life. bioXriv [Preprint] (2024), https://www.biorxiv.org/content/10.1101/2024.05.28.596244v1 [Accessed 5 December 2024].

[10] T. Rodrigues-Oliveira, A. Belmok, D. Vasconcellos, B. Schuster, C. M. Kyaw, Archaeal S-layers: Overview and current state of the art. Front. Microbiol. **8**, 2597 (2017). 29312266 10.3389/fmicb.2017.02597 PMC5744192

[11] A. Klingl, C. Pickl, J. Flechsler, “Archaeal cell walls” in Bacterial Cell Walls and Membranes, A. Kuhn, Ed. (Springer International Publishing, 2019), pp. 471–493. 10.1007/978-3-030-18768-2_14 31214995

[12] H. Huber , Ignicoccus gen. nov., a novel genus of hyperthermophilic, chemolithoautotrophic Archaea, represented by two new species, Ignicoccus islandicus sp nov and Ignicoccus pacificus sp nov. and Ignicoccus pacificus sp. nov. Int. J. Syst. Evol. Microbiol. **50**, 2093–2100 (2000). 11155984 10.1099/00207713-50-6-2093

[13] H. Huber, U. Küper, S. Daxer, R. Rachel, The unusual cell biology of the hyperthermophilic Crenarchaeon Ignicoccus hospitalis. Antonie van Leeuwenhoek **102**, 203–219 (2012). 22653377 10.1007/s10482-012-9748-5

[14] O. V. Golyshina , Oxyplasma meridianum gen. nov., sp. nov., an extremely acidophilic organotrophic member of the order Thermoplasmatales. Int. J. Syst. Evol. Microbiol. **74**, 006499 (2024). 39190454 10.1099/ijsem.0.006499 PMC11349054

[15] A. Klingl, S-layer and cytoplasmic membrane—exceptions from the typical archaeal cell wall with a focus on double membranes. Front. Microbiol. **5**, 624 (2014). 25505452 10.3389/fmicb.2014.00624 PMC4243693

[16] A. Fioravanti, M. Mathelie-Guinlet, Y. F. Dufrêne, H. Remaut, The Bacillus anthracis S-layer is an exoskeleton-like structure that imparts mechanical and osmotic stabilization to the cell wall. PNAS Nexus **1**, pgac121 (2022). 36714836 10.1093/pnasnexus/pgac121 PMC9802277

[17] A. von Kügelgen, V. Alva, T. A. M. Bharat, Complete atomic structure of a native archaeal cell surface. Cell Rep. **37**, 110052 (2021). 34818541 10.1016/j.celrep.2021.110052 PMC8640222

[18] L. Li , Enhanced glycosylation of an S-layer protein enables a psychrophilic methanogenic archaeon to adapt to elevated temperatures in abundant substrates. FEBS Lett. **594**, 665–677 (2020). 31665542 10.1002/1873-3468.13650

[19] K. F. Jarrell , N-linked glycosylation in Archaea: A structural, functional, and genetic analysis. Microbiol. Mol. Biol. Rev. **78**, 304–341 (2014). 24847024 10.1128/MMBR.00052-13 PMC4054257

[20] S. F. Koval, S. H. Hynes, Effect of paracrystalline protein surface layers on predation by Bdellovibrio bacteriovorus. J. Bacteriol. **173**, 2244–2249 (1991). 2007549 10.1128/jb.173.7.2244-2249.1991 PMC207774

[21] P.-N. Li , Nutrient transport suggests an evolutionary basis for charged archaeal surface layer proteins. ISME J. **12**, 2389–2402 (2018). 29899515 10.1038/s41396-018-0191-0 PMC6155111

[22] A. von Kügelgen , Membraneless channels sieve cations in ammonia-oxidizing marine archaea. Nature **630**, 230–236 (2024). 38811725 10.1038/s41586-024-07462-5 PMC11153153

[23] L. Gambelli , Architecture and modular assembly of Sulfolobus S-layers revealed by electron cryotomography. Proc. Natl. Acad. Sci. **116**, 25278–25286 (2019). 31767763 10.1073/pnas.1911262116 PMC6911244

[24] L. Gambelli , Structure of the two-component S-layer of the archaeon Sulfolobus acidocaldarius. eLife **13**, e84617 (2024). 38251732 10.7554/eLife.84617 PMC10903991

[25] A. Veith , Acidianus, Sulfolobus and Metallosphaera surface layers: Structure, composition and gene expression. Mol. Microbiol. **73**, 58–72 (2009). 19522740 10.1111/j.1365-2958.2009.06746.x

[26] K. A. Taylor, J. F. Deatherage, L. A. Amos, Structure of the S-layer of Sulfolobus acidocaldarius. Nature **299**, 840–842 (1982).

[27] D. W. Grogan, Organization and interactions of cell envelope proteins of the extreme thermoacidophile Sulfolobus acidocaldarius. Can. J. Microbiol. **42**, 1163–1171 (1996).

[28] E. Peyfoon , The S-layer glycoprotein of the crenarchaeote Sulfolobus acidocaldarius is glycosylated at multiple sites with chitobiose-linked N-glycans. Archaea **2010**, 754101 (2010). 20936123 10.1155/2010/754101 PMC2948927

[29] U. Zähringer, H. Moll, T. Hettmann, Y. A. Knirel, G. Schäfer, Cytochrome b558/566 from the archaeon Sulfolobus acidocaldarius has a unique Asn-linked highly branched hexasaccharide chain containing 6-sulfoquinovose. Eur. J. Biochem. **267**, 4144–4149 (2000). 10866817 10.1046/j.1432-1327.2000.01446.x

[30] C. J. Comerci , Topologically-guided continuous protein crystallization controls bacterial surface layer self-assembly. Nat. Commun. **10**, 2731 (2019). 31227690 10.1038/s41467-019-10650-x PMC6588578

[31] P. Oatley, J. A. Kirk, S. Ma, S. Jones, R. P. Fagan, Spatial organization of Clostridium difficile S-layer biogenesis. Sci. Rep. **10**, 14089 (2020). 32839524 10.1038/s41598-020-71059-x PMC7445750

[32] M. F. Abdul-Halim , Lipid anchoring of archaeosortase substrates and midcell growth in haloarchaea. mBio **11**, e00349-20 (2020). 32209681 10.1128/mBio.00349-20 PMC7157517

[33] M. Herdman , Cell cycle dependent coordination of surface layer biogenesis in Caulobacter crescentus. Nat. Commun. **15**, 3355 (2024). 38637514 10.1038/s41467-024-47529-5 PMC11026435

[34] A. W. Bisson-Filho , Treadmilling by FtsZ filaments drives peptidoglycan synthesis and bacterial cell division. Science **355**, 739–743 (2017). 28209898 10.1126/science.aak9973 PMC5485650

[35] D. Joseleau-Petit, J.-C. Liébart, J. A. Ayala, R. D’Ari, Unstable Escherichia coli L forms revisited: Growth requires peptidoglycan synthesis. J. Bacteriol. **189**, 6512–6520 (2007). 17586646 10.1128/JB.00273-07 PMC2045188

[36] A. Walther, J. Wendland, Septation and cytokinesis in fungi. Fungal Genet. Biol. **40**, 187–196 (2003). 14599886 10.1016/j.fgb.2003.08.005

[37] A. A. Pulschen , Live imaging of a hyperthermophilic archaeon reveals distinct roles for two ESCRT-III homologs in ensuring a robust and symmetric division. Curr. Biol. **30**, 2852–2859.e4 (2020). 32502411 10.1016/j.cub.2020.05.021 PMC7372223

[38] M. Wagner , Versatile genetic tool box for the crenarchaeote sulfolobus acidocaldarius. Front. Microbiol. **3**, 214 (2012). 22707949 10.3389/fmicb.2012.00214 PMC3374326

[39] C. Zhang , Cell structure changes in the hyperthermophilic crenarchaeon sulfolobus islandicus lacking the S-layer. mBio **10**, e01589-19 (2019). 31455649 10.1128/mBio.01589-19 PMC6712394

[40] I. A. Zink , CRISPR-mediated gene silencing reveals involvement of the archaeal S-layer in cell division and virus infection. Nat. Commun. **10**, 4797 (2019). 31641111 10.1038/s41467-019-12745-x PMC6805947

[41] C. Zhang, A. P. R. Phillips, R. L. Wipfler, G. J. Olsen, R. J. Whitaker, The essential genome of the crenarchaeal model Sulfolobus islandicus. Nat. Commun. **9**, 4908 (2018). 30464174 10.1038/s41467-018-07379-4 PMC6249222

[42] A. Krogh, B. Larsson, G. von Heijne, E. L. L. Sonnhammer, Predicting transmembrane protein topology with a hidden markov model: Application to complete genomes. J. Mol. Biol. **305**, 567–580 (2001). 11152613 10.1006/jmbi.2000.4315

[43] L. Käll, A. Krogh, E. L. L. Sonnhammer, Advantages of combined transmembrane topology and signal peptide prediction–the Phobius web server. Nucleic Acids Res. **35**, W429–432 (2007). 17483518 10.1093/nar/gkm256 PMC1933244

[44] J. Jumper , Highly accurate protein structure prediction with AlphaFold. Nature **596**, 583–589 (2021). 34265844 10.1038/s41586-021-03819-2 PMC8371605

[45] M. Varadi , AlphaFold protein structure database in 2024: Providing structure coverage for over 214 million protein sequences. Nucleic Acids Res. **52**, D368–D375 (2024). 37933859 10.1093/nar/gkad1011 PMC10767828

[46] J. Abramson , Accurate structure prediction of biomolecular interactions with AlphaFold 3. Nature **630**, 493–500 (2024). 38718835 10.1038/s41586-024-07487-w PMC11168924

[47] A. M. Lau , Exploring structural diversity across the protein universe with The Encyclopedia of Domains. Science **386**, eadq4946 (2024). 39480926 10.1126/science.adq4946 PMC7618865

[48] S. Bittrich, J. Segura, J. M. Duarte, S. K. Burley, Y. Rose, RCSB protein data bank: Exploring protein 3D similarities via comprehensive structural alignments. Bioinformatics **40**, btae370 (2024). 38870521 10.1093/bioinformatics/btae370 PMC11212067

[49] Y. Zhang, J. Skolnick, TM-align: A protein structure alignment algorithm based on the TM-score. Nucleic Acids Res. **33**, 2302–2309 (2005). 15849316 10.1093/nar/gki524 PMC1084323

[50] A. Sogues , Cryo-EM structure and polar assembly of the PS2 S-layer of Corynebacterium glutamicum. bioXriv [Preprint] (2024), 10.1101/2024.09.05.611363 (Accessed 5 December 2024). PMC12337289 40729392

[51] P. Simonin, C. Lombard, A. Huguet, A. Kish, Improved Isolation of SlaA and SlaB S-layer proteins in Sulfolobus acidocaldarius. Extremophiles **24**, 673–680 (2020). 32494965 10.1007/s00792-020-01179-9

[52] A.-C. Lindås, E. A. Karlsson, M. T. Lindgren, T. J. G. Ettema, R. Bernander, A unique cell division machinery in the Archaea. Proc. Natl. Acad. Sci. U.S.A. **105**, 18942–18946 (2008). 18987308 10.1073/pnas.0809467105 PMC2596248

[53] R. Y. Samson, T. Obita, S. M. Freund, R. L. Williams, S. D. Bell, A role for the ESCRT system in cell division in archaea. Science **322**, 1710–1713 (2008). 19008417 10.1126/science.1165322 PMC4121953

[54] J. Liu , Functional assignment of multiple ESCRT-III homologs in cell division and budding in Sulfolobus islandicus. Mol. Microbiol. **105**, 540–553 (2017). 28557139 10.1111/mmi.13716

[55] R. Y. Samson , Molecular and structural basis of ESCRT-III recruitment to membranes during archaeal cell division. Mol. Cell **41**, 186–196 (2011). 21255729 10.1016/j.molcel.2010.12.018 PMC3763469

[56] L. Harker-Kirschneck , Physical mechanisms of ESCRT-III-driven cell division. Proc. Natl. Acad. Sci. U.S.A. **119**, e2107763119 (2022). 34983838 10.1073/pnas.2107763119 PMC8740586

[57] T. Minamino, K. Namba, Self-assembly and type III protein export of the bacterial flagellum. J. Mol. Microbiol. Biotechnol. **7**, 5–17 (2004). 15170399 10.1159/000077865

[58] M. A. Lauffer, Entropy-driven processes in biology. Mol. Biol. Biochem. Biophys. **20**, 1–264 (1975), 10.1007/978-3-642-80869-2. 785232

[59] G. Chiesa, S. Kiriakov, A. S. Khalil, Protein assembly systems in natural and synthetic biology. BMC Biol. **18**, 35 (2020). 32216777 10.1186/s12915-020-0751-4 PMC7099826

[60] S. Saad, D. F. Jarosz, Protein self-assembly: A new frontier in cell signaling. Curr. Opin. Cell Biol. **69**, 62–69 (2021). 33493989 10.1016/j.ceb.2020.12.013 PMC8058241

[61] F. Frey, U. S. Schwarz, Coat stiffening can explain invagination of clathrin-coated membranes. Phys. Rev. E **110**, 064403 (2024). 39916158 10.1103/PhysRevE.110.064403

[62] J. A. Ybe , Clathrin self-assembly is mediated by a tandemly repeated superhelix. Nature **399**, 371–375 (1999). 10360576 10.1038/20708

[63] I. Raote, S. Saxena, V. Malhotra, Sorting and export of proteins at the endoplasmic reticulum. Cold Spring Harb. Perspect. Biol. **15**, a041258 (2023). 35940902 10.1101/cshperspect.a041258 PMC10153803

[64] N. Bhattacharya, J. O’Donnell, S. M. Stagg, The structure of the Sec13/31 COPII Cage bound to Sec23. J. Mol. Biol. **420**, 324–334 (2012). 22543240 10.1016/j.jmb.2012.04.024 PMC3377835

[65] T. Kirchhausen, D. Owen, S. C. Harrison, Molecular structure, function, and dynamics of Clathrin-mediated membrane traffic. Cold Spring Harb. Perspect. Biol. **6**, a016725 (2014). 24789820 10.1101/cshperspect.a016725 PMC3996469

[66] S. M. Sweitzer, J. E. Hinshaw, Dynamin undergoes a GTP-dependent conformational change causing vesiculation. Cell **93**, 1021–1029 (1998). 9635431 10.1016/s0092-8674(00)81207-6

[67] M. C. S. Lee , Sar1p N-terminal helix initiates membrane curvature and completes the fission of a COPII vesicle. Cell **122**, 605–617 (2005). 16122427 10.1016/j.cell.2005.07.025

[68] G. Tagiltsev, C. A. Haselwandter, S. Scheuring, Nanodissected elastically loaded clathrin lattices relax to increased curvature. Sci. Adv. **7**, eabg9934 (2021). 34389539 10.1126/sciadv.abg9934 PMC8363152

[69] K. A. Sochacki , The structure and spontaneous curvature of clathrin lattices at the plasma membrane. Dev. Cell **56**, 1131–1146.e3 (2021). 33823128 10.1016/j.devcel.2021.03.017 PMC8081270

[70] X. Zhou , Mechanical crack propagation drives millisecond daughter cell separation in Staphylococcus aureus. Science **348**, 574–578 (2015). 25931560 10.1126/science.aaa1511 PMC4864021

[71] R. Jaenicke, R. Welsch, M. Sára, U. B. Sleytr, Stability and self-assembly of the S-layer protein of the cell wall of Bacillus stearothermophilus. Biol. Chem. Hoppe Seyler **366**, 663–670 (1985). 4041240 10.1515/bchm3.1985.366.2.663

[72] D. Pum, U. B. Sleytr, Monomolecular reassembly of a crystalline bacterial cell surface layer (S-layer) on untreated and modified silicon surfaces. Supramol. Sci. **2**, 193–197 (1995).

[73] S.-H. Shin , Direct observation of kinetic traps associated with structural transformations leading to multiple pathways of S-layer assembly. Proc. Natl. Acad. Sci. U.S.A. **109**, 12968–12973 (2012). 22822216 10.1073/pnas.1201504109 PMC3420203

[74] A. E. Lopez, S. Moreno-Flores, D. Pum, U. B. Sleytr, J. L. Toca-Herrera, Surface dependence of protein nanocrystal formation. Small **6**, 396–403 (2010). 19943246 10.1002/smll.200901169

[75] M. Herdman , High-resolution mapping of metal ions reveals principles of surface layer assembly in Caulobacter crescentus cells. Structure **30**, 215–228.e5 (2022). 34800371 10.1016/j.str.2021.10.012 PMC8828063

[76] T. D. Brock, K. M. Brock, R. T. Belly, R. L. Weiss, Sulfolobus: A new genus of sulfur-oxidizing bacteria living at low pH and high temperature. Arch. Mikrobiol. **84**, 54–68 (1972). 4559703 10.1007/BF00408082

[77] A. Cezanne, B. Hoogenberg, B. Baum, Probing archaeal cell biology: Exploring the use of dyes in the imaging of Sulfolobus cells. Front. Microbiol. **14**, 1233032 (2023). 37731920 10.3389/fmicb.2023.1233032 PMC10508906

[78] N. van der Kolk , Identification of XylR, the activator of arabinose/xylose inducible regulon in sulfolobus acidocaldarius and its application for homologous protein expression. Front. Microbiol. **11**, 1066 (2020). 32528450 10.3389/fmicb.2020.01066 PMC7264815

[79] A. von Kügelgen , Interdigitated immunoglobulin arrays form the hyperstable surface layer of the extremophilic bacterium Deinococcus radiodurans. Proc. Natl. Acad. Sci. **120**, e2215808120 (2023). 37043530 10.1073/pnas.2215808120 PMC10120038

[80] B. Isbilir, A. Yeates, V. Alva, T. A. M. Bharat, Mapping the ultrastructural topology of the corynebacterial cell surface. bioXriv [Preprint] (2024), https://www.biorxiv.org/content/10.1101/2024.09.05.611374v1 [Accessed 26 December 2024]. 10.1371/journal.pbio.3003130 PMC12021427 40233127

[81] P. Thévenaz, U. E. Ruttimann, M. Unser, A pyramid approach to subpixel registration based on intensity. IEEE Trans. Image Process. **7**, 27–41 (1998). 18267377 10.1109/83.650848

[82] H. Mary, G. J. Brouhard, Kappa (κ): Analysis of curvature in biological image data using B-splines. bioXriv [Preprint] (2019), https://www.biorxiv.org/content/10.1101/852772v1 [Accessed 6 December 2024].

