# Supplementary material for: A self-assembling surface layer flattens the cytokinetic furrow to aid cell division in an archaeon
Source: Proc Natl Acad Sci U S A. 2025 Jun 18;122(25):e2501044122. doi: 10.1073/pnas.2501044122 (PMC12207459; doi:10.1073/pnas.2501044122)
Supplement: Supplementary file 1 — Appendix 01 (PDF) [file pnas.2501044122.sapp.pdf]

PNAS

Supporting Information for
A self-assembling surface layer flattens the cytokinetic furrow to aid cell division in an archaeon

Sherman Foo[a], Ido Caspy[a,1], Alice Cezanne[a,1], Tanmay A. M. Bharat[a] & Buzz Baum[a,2]

[a]Medical Research Council Laboratory of Molecular Biology, Cambridge CB2 0QH, United Kingdom
[1]These authors contributed equally to this work
[2]To whom correspondence may be addressed. Email: bbaum@mrc-lmb.cam.ac.uk

This PDF file includes:

Supplementary Figures S1 to S4
Legends for Supplementary Movies S1 to S4
Extended Materials and Methods
Supplementary Tables S1 and S2
References

Other supporting materials for this manuscript include the following:

Supplementary Movies S1 to S4

Supplementary Figures

A

Strain	Position	Mutation	Annotation	Gene	Description
Δ*slaA*	1,824,316	T>TC	Insertion	Non-coding	-
	2,199,897	Δ4275 bp	Deletion	Saci2355	Surface layer protein A
Δ*slaB*	1,613,112	T>TA	Insertion: Stop (TAA>TAAA)	Saci1845	Conserved protein
	2,198,399	Δ1428 bp	Deletion	Saci2354	Surface layer protein B
Δ*slaAB*	2,198,399	Δ5772 bp	Deletion	Saci2354, Saci2355	Surface layer protein B, Surface layer protein A
Δ*saci1846*	1,613,165	Δ3195 bp	Deletion	Saci1846	Thermopsin-like protein
Δ*slaB* Δ*saci1846*	1,613,165	Δ3195 bp	Deletion	Saci1846	Thermopsin-like protein
	2,198,399	Δ1428 bp	Deletion	Saci2354	Surface layer protein B

B

No.	Gene	Gene ID	Description
1.	*slaA*	Saci2355	S-layer protein A
2.	*albA*	Saci1322	DNA/RNA-binding protein Alba
3.		Saci0641	Peptidyl-prolyl cis-trans isomerase
4.	*rps3*	Saci0591	Small ribosomal subunit protein uS3
5.		Saci1386	SHSP domain-containing protein
6.	*thsB*	Saci0666	Thermosome subunit beta
7.	*tuf*	Saci0685	Elongation factor 1-alpha
8.	*mat*	Saci0798	S-adenosylmethionine synthase
9.	*thsA*	Saci1401	Thermosome subunit alpha
10.		Saci1749	Band 7 domain-containing protein
11.	*rpl12*	Saci1456	Large ribosomal subunit protein P1
12.		Saci0922	Small heat shock protein
13.	*srp54*	Saci1310	Signal recognition particle 54 kDa protein
14.	*tip49*	Saci0552	DNA helicase
15.		Saci1140	Conserved T+S rich domain protein
16.	*atpD*	Saci1550	V-type ATP synthase subunit D
17.		Saci1546	V-type ATP synthase subunit F
18.		Saci0994	Ribosomal protein S6 modification protein
19.	*pfdB*	Saci0605	Prefoldin subunit beta
20.	*slaB*	Saci2354	S-layer protein B
21.		Saci1788	Conserved Archaeal protein
22.		Saci0713	Threonine ammonia-lyase
23.		Saci0064	DNA-binding protein 7d
24.		Saci0331	Pyridine nucleotide-disulphide oxidoreductase
25.	*gap*	Saci1356	Glyceraldehyde-3-phosphate dehydrogenase
26.		Saci1764	ABC transporter
27.		Saci1585	Radical SAM core domain-containing protein
28.	*rps10*	Saci0684	Small ribosomal subunit protein uS10
29.	*psmB2*	Saci0909	Proteasome subunit beta 2
30.		Saci1183	Conserved Archaeal membrane protein

C

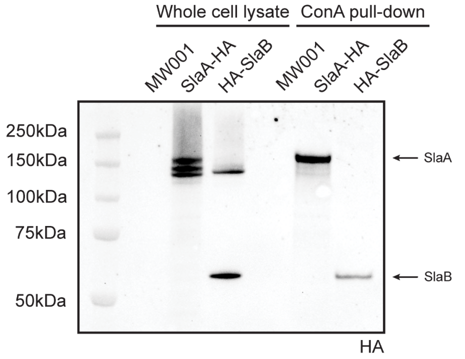

D

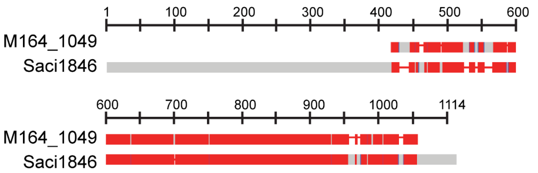

E

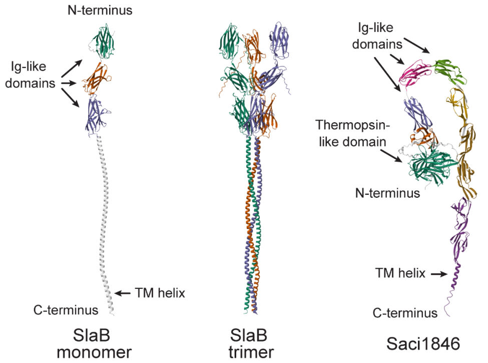

F

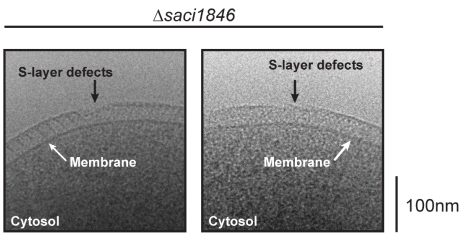

G

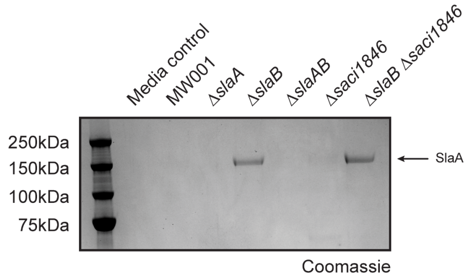

Figure S1. Validating S-layer mutants and their visualization with concanavalin A.

(**A**) Whole genome sequencing of the uracil auxotrophic background strain MW001 and the respective mutants generated in this study was performed. A summary of mutations identified compared to MW001 is presented here. (**B**) Top 30 hits from mass spectrometry analysis of proteins pulled down from wild-type *S. acidocaldarius* DSM639 lysates using ConA magnetic beads. (**C**) Whole cell lysates of MW001 and HA-tagged SlaA or SlaB cells were incubated with magnetic ConA beads to assess ConA binding to the highly glycosylated S-layer proteins. Western blot of input cell lysates and the ConA pull-down assay shows successful pull-down of the HA-tagged SlaA and SlaB proteins, visualised using anti-HA antibodies. (**D**) Protein sequence alignment of M164_1049 and Saci1846 using the NCBI Multiple Sequence Alignment Viewer. Alignment positions are coloured red indicating highly conserved positions, blue indicating lower conservation and grey indicating unaligned residues. (**E**) AlphaFold models of SlaB, SlaB trimer and Saci1846. Structural domains classified using The Encyclopedia of

Domains on the AlphaFold Protein Structure Database are depicted in different colours for the SlaB monomer and Saci1846, while each SlaB monomers are depicted in different colours in its trimeric form. (**F**) Minor S-layer defects are observed in the Δ*saci1846* mutants, as shown in these representative cryo-EM images. (**G**) Coomassie-stained gel of TCA precipitated proteins from the growth medium after culturing of the indicated mutant strains.

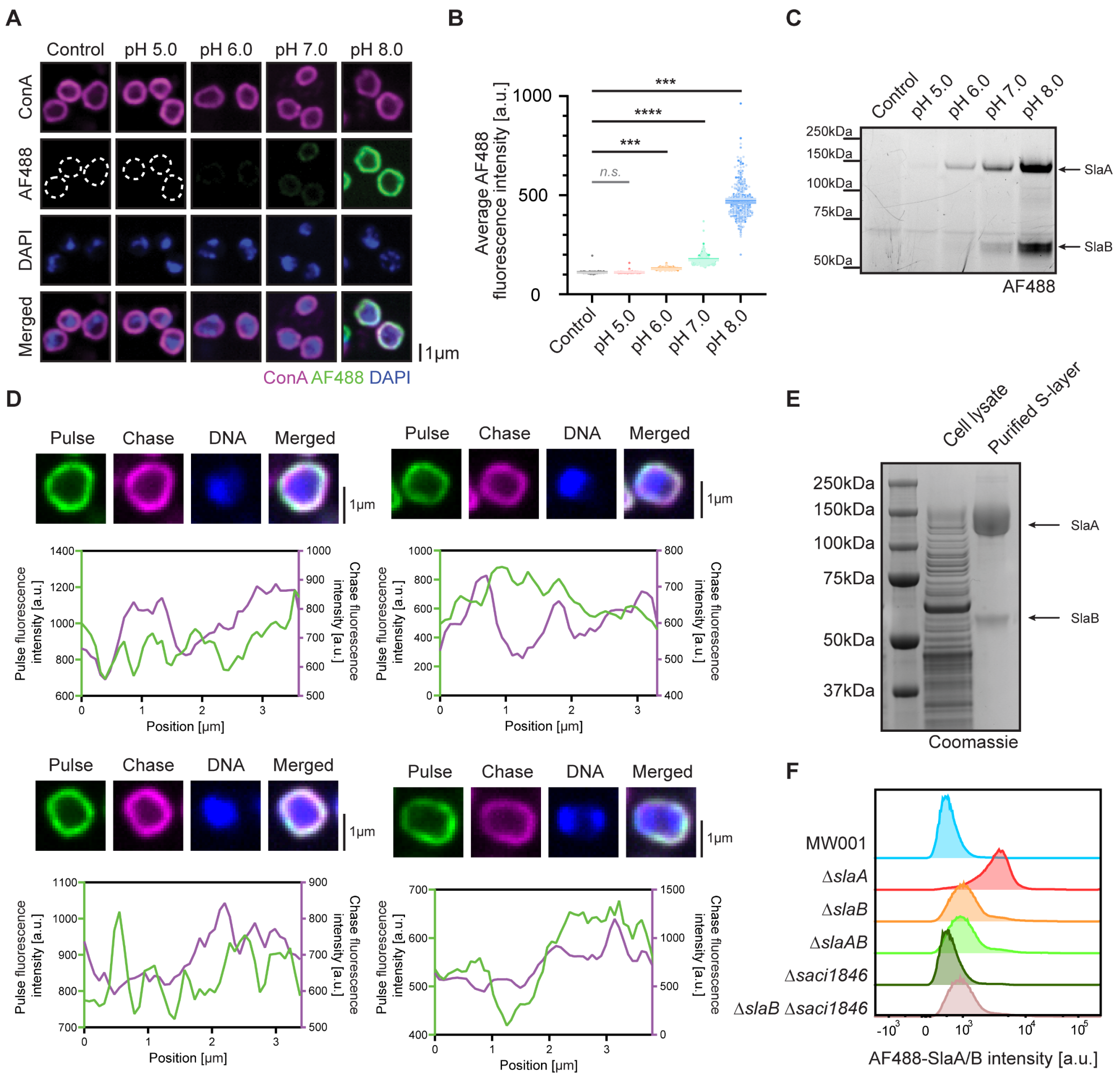

Figure S2. Labelling of the *S. acidocaldarius* cell surface with NHS ester dyes and exogenous S-layer proteins.

(**A**) MW001 cells were labelled with Alexa Fluor 488 (AF488) NHS ester in Brock medium (control, pH 3.0), or in buffers at a range of different pH. Shown here are representative immunofluorescence images. (**B**) Quantification of labelling efficiency at different pH was performed by measuring average fluorescence intensities at the cell surface (N=3, n=100 cells each). Each biological replicate is represented by a different shade of colour and the means indicated (Control: 110.3 ±1.51 a.u.; pH 5.0: 111.3 ±0.410 a.u.; pH 6.0: 129.7 ±1.41 a.u.; pH 7.0: 173.9 ±2.15 a.u.; pH 8.0: 472.7 ±8.42 a.u.). *P*-values were derived using Welch's *t*-test (N=3, ****p* ≤0.001; *****p* ≤0.0001; *n.s.*, no significance). (**C**) SDS-PAGE gel of whole cell fractions following labelling at different pH, showing strong labelling at basic pH of two major protein bands corresponding to the molecular weights of SlaA and SlaB. Labelled proteins were visualised using the Alexa 488 function (UV 520/30) on a BioRad ChemiDoc MP imaging system. (**D**) MW001 cells were pulse labelled using AF488 (green) and chase labelled using Alexa Fluor 647 (magenta) NHS esters based dyes. Shown here are representative cells, including a dividing cell (bottom right), together with the plot profile of the respective fluorescence intensities below each example. (**E**) Coomassie-stained gel showing purified wild-type SlaA and SlaB extracted from wild-type *S. acidocaldarius* DSM639 cultures. (**F**) Purified S-layer proteins labelled with AF488 NHS ester dye were incubated with S-layer mutants. Shown here are representative flow cytometry histograms of AF488-SlaA/B intensities in each mutant background, representing the efficiency of labelling these mutants with exogenous S-layer proteins (N=3, n=2.5x10^5 events each).

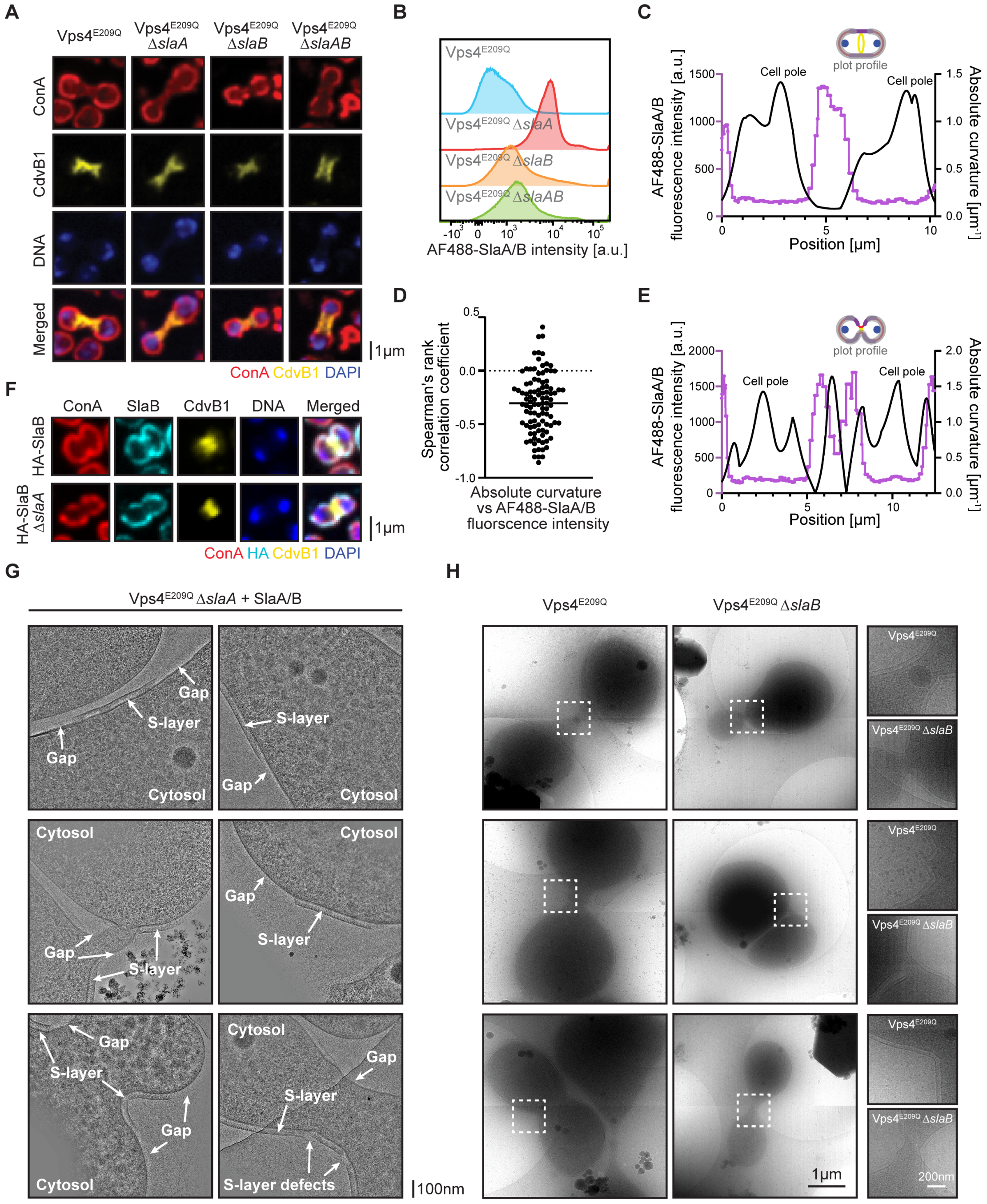

Figure S3. Labelling of S-layer mutants expressing Vps4^{E209Q} arrested at cell division with exogenous S-layer proteins.

(**A**) Representative single-plane spinning-disk confocal images of MW001 and the S-layer mutants expressing the Walker B dominant negative mutant Vps4^{E209Q} shows cells arrested at division. (**B**) Purified S-layer proteins labelled with Alexa Fluor 488 (AF488) NHS ester dye were incubated with MW001 or the S-layer mutants overexpressing Vps4^{E209Q}. Shown here are representative flow cytometry histograms of AF488-SlaA/B intensities in each mutant background, representing the efficiency of labelling these mutants with exogenous S-layer proteins (N=3, n=2.5x10^5 events each). (**C**) Representative Kappa curvature analysis of a pre-constriction Vps4^{E209Q} Δ*slaA* cell showing the absolute point curvature (black) and the fluorescence intensity of exogenous S-layer protein (magenta) on the cell surface. A diagram showing the plot profile used indicated in grey is shown above. (**D**) Spearman's correlation coefficient between the absolute point curvature and the fluorescence intensity of exogenous S-layer proteins on the surface of pre-constriction Vps4^{E209Q} Δ*slaA* cells (n=100 cells). (**E**) Representative Kappa curvature analysis of a late constriction Vps4^{E209Q} Δ*slaA* cell showing

the absolute point curvature (black) and the fluorescence intensity of exogenous S-layer protein (magenta) on the cell surface. A diagram showing the plot profile used indicated in grey is shown above. (**F**) Representative single-plane spinning-disk confocal images of dividing MW001 and the Δ*slaA* mutant expressing HA-SlaB following 4h of arabinose induction. (**G**) Gallery of representative cryo-EM images of Vps4^{E209Q} Δ*slaA* mutants with exogenous unlabelled S-layer proteins added, showing the formation of lattices by the exogenous S-layer proteins on the cell surface via self-assembly. (**H**) Gallery of representative cryo-EM images of Vps4^{E209Q} and Vps4^{E209Q} Δ*slaB* mutants. Magnified images of the division bridges indicated by the white boxes in each example are shown on the right.

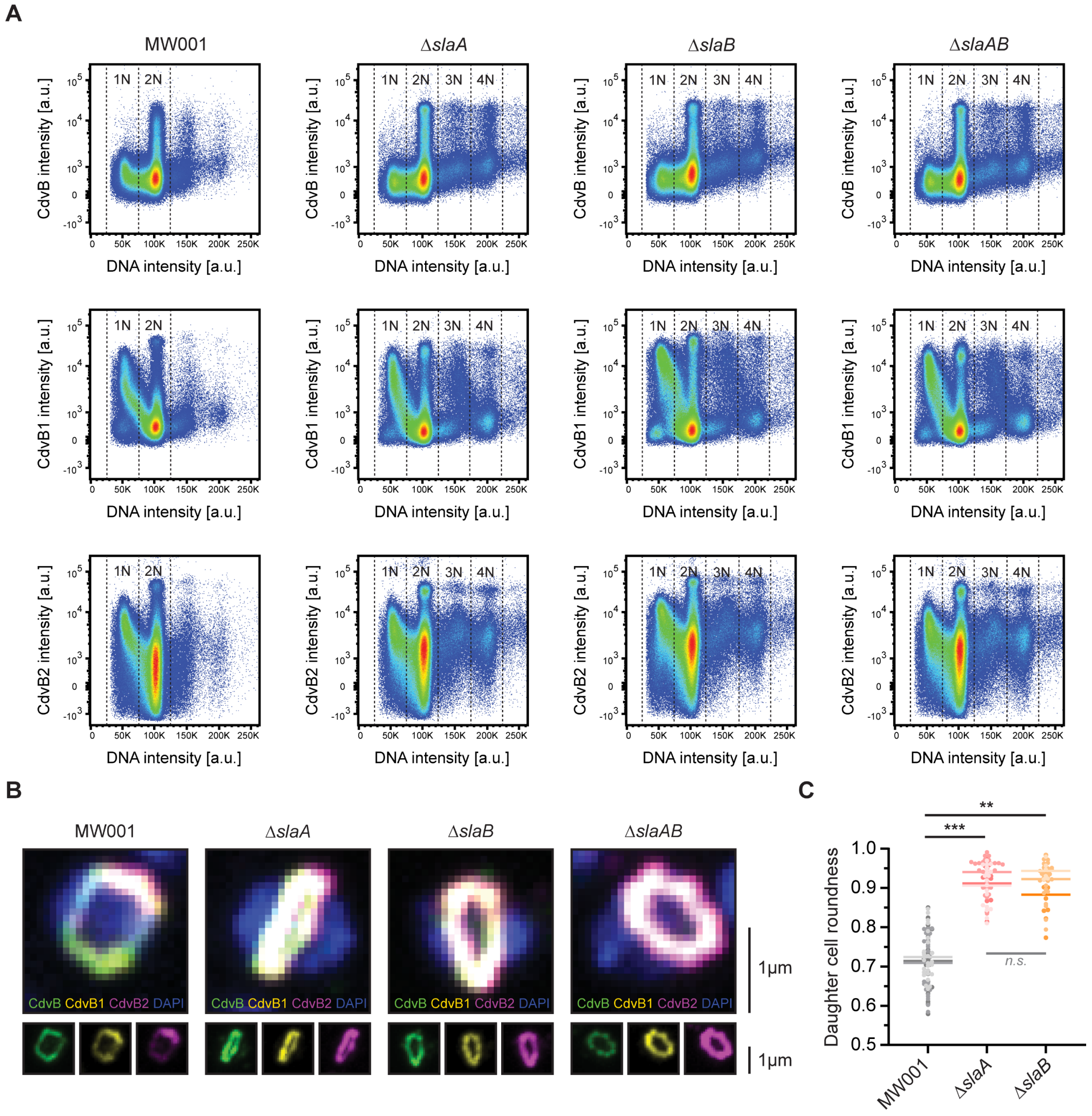

Figure S4. The ESCRT-III rings of S-layer mutants.

(**A**) Representative flow cytometry scatter plots of asynchronous cultures of MW001 and the S-layer mutants. CdvB (top), CdvB1 (middle) and CdvB2 (bottom) signals are plotted against DNA in each strain. Each spot represents a single cell, with the density gradient going from blue to red (N=3, n=5.0x10^5 events each). (**B**) Representative maximum intensity z-axis projections of MW001 and the S-layer mutants during mitosis shows formation of the ESCRT-III rings. Cells were stained for DNA with DAPI (blue), CdvB (green), CdvB1 (yellow) and CdvB2 (magenta). (**C**) Quantification of the roundness of daughter cells under live imaging conditions. Shown here are scatter plots with means indicated (MW001: 0.724 ±0.0064; Δ*slaA*: 0.919 ±0.015; Δ*slaB*: 0.916 ±0.025). *P*-values were derived using Welch's *t*-test (N=3, n ≥6 cells each; ****p* ≤0.001; ***p* ≤0.01).

Legends for Supplementary Movies

Movie S1. Cryo-ET of MW001 expressing Vps4^{E209Q}.

The S-layer is present with no visible gaps on the cell surface of MW001 expressing the Walker B dominant negative mutant Vps4^{E209Q} arrested at division. Scale bar represents 100nm.

Movie S2. Cryo-ET of MW001 expressing Vps4^{E209Q}.

The S-layer is present with no visible gaps on the cell surface of MW001 expressing the Walker B dominant negative mutant Vps4^{E209Q} arrested at division. Scale bar represents 100nm.

Movie S3. Cryo-ET of Δ*slaB* mutants expressing Vps4^{E209Q}.

The S-layer patches present in Δ*slaB* mutants preferentially localizes to the division bridge in Δ*slaB* cells expressing the Walker B dominant negative mutant Vps4^{E209Q} arrested at division. Scale bar represents 100nm.

Movie S4. Cryo-ET of MW001 expressing Vps4^{E209Q}.

The S-layer patches present in Δ*slaB* mutants preferentially localizes to the division bridge in Δ*slaB* cells expressing the Walker B dominant negative mutant Vps4^{E209Q} arrested at division. Scale bar represents 100nm.

Extended Materials and Methods

Electrocompetent *S. acidocaldarius* cells

Electrocompetent cells were made by growing 50mL of the strain of interest to an OD_{595} of 0.20-0.30. The culture was cooled down on ice, followed by collection via centrifugation at 2500rcf for 10min at 4°C. The cell pellet was washed twice with 30mL of ice-cold 20mM sucrose. Next, the pellet was resuspended in 1mL of ice-cold 20mM sucrose and transferred to a fresh microcentrifuge tube. Cells were centrifuged at 2500rcf for 10min at 4°C, resuspended with ice-cold 20mM sucrose to a final theoretical OD_{595} of 10, split into 50µL aliquots and stored at -70°C.

Molecular genetics

Deletion mutants were generated as follows. Briefly, the upstream and downstream regions of a gene of interest was cloned via restriction digest into pSVA406 (2). Positive clones were identified via miniprep and analytical digestion. Plasmids of interest were methylated by transformation into *E. coli* ER1821, and subsequently purified using a GeneJET plasmid miniprep kit (ThermoFisher Scientific, K0503).

A 50µL aliquot of electrocompetent *S. acidocaldarius* was thawed on ice. 200ng of methylated plasmid DNA was next added and the mixture transferred to a 1mm Gene Pulser Electroporation Cuvette (BioRad, 1652089). Electroporation was performed at 2000V, 25µF and 600Ω. 400µL of pH 5.0 Brock medium without sucrose supplemented was then added and the cells incubated in a fresh microcentrifuge tube at 75°C for 60min for recovery. 150µL of cells were then plated on solid Gelrite-Brock plates for 5 days at 75°C. Transformed colonies were next selected and inoculated in 10mL of liquid Brock medium supplemented with 0.1% (w/v) NZ-amine and 0.2% (w/v) sucrose and incubated for 2 days at 75°C. Positive clones were identified through genomic DNA extraction and genotyping of the overlapping regions of the locus of interest. Genomic DNA extraction was performed by first pelleting 500µL of cells through centrifugation at maximum speed for 30s. Next, 20µL of 0.1M NaOH was added and the pellet dissolved, followed by addition of 80µL of 0.1M Tris-HCL pH 8.0.

20µL of strains containing the inserts were next streaked onto Gelrite-Brock plates supplemented with 4µg/mL uracil and 100µg/mL 5-fluoroorotic acid (5-FOA; Zymo Research, P9001-1). Following 5 days of incubation at 75°C, colonies were selected and incubated in 10mL of liquid Brock medium supplemented with 0.1% (w/v) NZ-amine, 0.2% (w/v) sucrose, 4µg/mL uracil and 100µg/mL 5-FOA for 2 days at 75°C. Positive clones were identified through genomic DNA extraction and genotyping of the overlapping regions of the locus of interest. Positive clones were frozen in Brock medium containing 50% glycerol (v/v) and stored at -70°C.

Whole genome sequencing

20mL of OD_{595} 0.25 cells were pelleted and genomic DNA extraction performed by phenol/chloroform extraction. Cell pellets were first resuspended in 250µL of 10mM Tris-HCl pH 8.0, 1mM EDTA, 150mM NaCl, 0.1% TritonX-100 buffer and incubated at room temperature for 10min. Next, 250µL of phenol:chloroform:isoamyl alcohol (25:24:1, v/v) (Invitrogen, 15593031) was added and the mixture vortexed for 30s. The mixture was next centrifuged for 5min at 16,000rcf at room temperature. Following the transfer of the upper aqueous phase to a fresh microcentrifuge tube, the phenol/chloroform extraction was repeated twice. 200µL of the final upper aqueous phase was then transferred to a fresh microcentrifuge tube. Next, 150µL of 5M ammonium acetate and 875µL of 100% ethanol was added and this mixture was stored at -20°C overnight for DNA precipitation. The precipitated DNA was then pelleted at 16,000rcf for 5min at 4°C, and the DNA pellet washed twice in ice-cold 70% ethanol. The ethanol was removed and the pellet air dried at room temperature for 10min. The DNA pellet was then resuspended in 50µL of nuclease free water and stored at 4°C.

DNA concentration was determined using a Qubit dsDNS BR assay (Invitrogen, Q33265) on a Qubit Flex Fluorometer (Invitrogen), following the manufacturer's instructions. Whole genome sequencing and genome assembly was performed by Genewiz (Azenta Life Sciences).

Cryo-EM and cryo-ET sample preparation, data collection and image analysis

Samples for cryo-EM and cryo-ET were prepared as follows. 2.5µL of the *S. acidocaldarius* cell culture were applied onto glow-discharged Quantifoil R3.5/1 Cu/Rh grids, blotted for 4-5 seconds with a blot force ranging from -10 to -4 after waiting 10 seconds and immediately plunge frozen into liquid ethane maintained at -178°C using a Vitrobot Mark IV (Thermo Fisher Scientific) cooled to 10°C with 100% chamber humidity.

Cryo-EM images were captured using a Glacios TEM (Thermo Fisher Scientific) operating at 200kV using a Falcon 3 (Thermo Fisher Scientific) direct electron detector. *S. acidocaldarius* MW001, Δ*slaA*, Δ*slaB* and Δ*slaAB* were imaged at a nominal magnification of 73,000 resulting in a 1.994Å pixel size, using a total dose of 40e^-/Å^2 within a defocus range of -6 to -10µm. Δ*saci1846* and Δ*saci1846* Δ*slaB* were imaged at a nominal magnification of 92,000 resulting in a 1.583Å pixel size, using a dose of 40e^-/Å^2 within a defocus range of -6 to -10µm. Vps4^{E209Q} Δ*slaA* with exogenously added SlaA were imaged at a nominal magnification of 57,000 resulting in a 2.545Å pixel size using a dose of 40e^-/Å^2 within a defocus range of -6 to -10µm. Lower magnification images in Fig. S3H have pixel size of 47.8Å.

Tilt-series data was acquired using a Krios G3 TEM (Thermo Fisher Scientific) operating at 300kV using a K3 (Gatan) direct electron detector equipped with a Quantum energy filter (Gatan) with a slit width of 20eV. Data collection was performed using SerialEM software (3) with the dose-symmetric scheme (4) at a nominal magnification of 26,000, resulting in a pixel size of 2.809Å in counting mode. Tilt images were acquired at a tilt range of ±60° with 3° tilt increment with a total dose of 121-122e^-/Å^2 and with defoci ranging from -4 to -7µm (Vps4^{E209Q}) or -7 to -11µm (Vps4^{E209Q}Δ*slaB*). Frames from tilt series movies were motion corrected using RELION (5) and tilt-images were aligned and SART filtered using AreTomo (6). Final tomograms were reconstructed using the RELION5 pipeline (7) with 4x binning resulting in a final pixel size of 11.236Å.

Immunofluorescence labelling

Fixed cells were first pelleted via centrifugation at 8000rcf for 3min to remove ethanol and washed twice in 1mL of phosphate-buffered saline supplemented with 0.2% Tween-20 (Sigma-Aldrich, P9416) and 3% Bovine Serum Albumin (Sigma-Aldrich, A7030) (PBSTA) for rehydration and blocking. Cells were then incubated overnight at room temperature in 200µL of PBSTA containing primary antibodies (Supplementary Table S2) with 400rpm agitation on an Eppendorf thermomixer. Following two washes in 1mL phosphate-buffered saline supplemented with 0.2% Tween-20 (PBST), 200µL of PBSTA containing secondary antibodies (Supplementary Table S2) were added for 2h at room temperature and 400rpm agitation on an Eppendorf thermomixer. Labelling of cell surface was performed by incubating with 5µg/mL concanavalin A conjugated to Alexa Fluor 647 (Thermo Fisher Scientific, C21421) during secondary antibody incubation. Following two washes in 1mL PBST, cells were resuspended in 200µL of PBST containing either 1µg/mL DAPI (4′,6-diamidino-2-phenylindole; Thermo Fisher Scientific, 62248) for imaging, or 2µM Hoechst (Thermo Fisher Scientific, 62249) for flow cytometry experiments.

Labelling of cell surface with NHS ester dyes

30mL of exponential growing *S. acidocaldarius* cells were first collected via centrifugation at 4000rcf for 5min. The cell pellet was washed once in 1mL of 50mM HEPES pH 8.0 buffer before being resuspended in 1mL of 50mM HEPES pH 8.0. Pulse labelling was performed by addition of 3µL of a 1mg/mL stock of Alexa Fluor 488 NHS succinimidyl ester dye (Invitrogen, A20000) in DMSO to the solution and incubated in an Eppendorf thermomixer at room temperature for 30min with 500rpm shaking. Next, the cells were washed once in 1mL of 50mM HEPES pH 8.0 and once in 1mL of pre-warmed Brock medium. The cell pellet was resuspended in Brock medium and diluted into 25mL of pre-warmed Brock medium and allowed to grow for 2h. The process was then repeated with Alexa Fluor 647 NHS succinimidyl ester dye (Invitrogen, A20009) for chase labelling, and cells were fixed in ethanol as described previously.

Optimization of labelling with NHS ester dyes was performed as described above, using Alexa Fluor 488 NHS succinimidyl ester dye (Invitrogen, A20000) in different buffers. 50mM Citrate buffer at pH 5.0 or pH 6.0, or 50mM HEPES buffer at pH 7.0 or 8.0 was used for the wash steps

and incubation with the dye. Further analysis of NHS ester dye labelled proteins was performed via SDS-PAGE. 10mL of OD_{595} 0.1 of labelled cells was collected via centrifugation and resuspended in 200µL of 1x NuPAGE LDS sample buffer (Thermo Fisher Scientific, NP0007) containing 10% β-mercaptoethanol and incubated at 98°C for 5min. Following a brief centrifugation, samples were loaded and ran on a NuPAGE 4 to 12% Bis-Tris gels (Invitrogen, NP0321BOX) at a constant 150V for 60-80min using MOPS running buffer. The gel was imaged directly on a BioRad ChemiDoc MP imaging system using the Alexa 488 function (UV 520/30).

S-layer purification

1L of wild-type *S. acidocaldarius* DSM639 was grown to an OD_{595} of 0.8, following standard cell husbandry protocols as described above. Cells were centrifuged at 4000rcf for 30min using four 250mL large volume centrifuge tubes (Corning, CLS430776) and the supernatant was discarded. The cell pellets were resuspended in water and transferred to two 50mL falcon tubes before being centrifuged at 4000rcf for 30min. The supernatant was discarded and the falcon tubes containing the cell pellets were stored at -70°C until further use.

S-layer purification was performed as described previously (8, 9), with minor modifications for compatibility with benchtop centrifugation. Each cell pellet was first thawed and resuspended in 40mL of 10mM HEPES buffer pH 7.0 containing 2mM EDTA and 1mM PMSF (HEPES wash buffer), supplemented with 600µL of 10% SDS to obtain a final concentration of 0.15% SDS. 100µg/µL DNaseI (ITW reagents, A3778.0100) and 4mM $MgCl_2$ was added and the mixture incubated for 1h at 37°C. An additional 8mL of 10% SDS was added to bring the final SDS concentration to 2% and the mixture was left overnight on a roller mixer at room temperature. The mixture was next centrifuged for 1h at room temperature at 4000rcf and the supernatant discarded. The pellet was washed with 30mL of HEPES wash buffer +2% SDS for 1h at 75°C with 200rpm shaking in the incubator, followed by centrifugation for 1h at room temperature at 4000rcf and the supernatant discarded. This wash was repeated for a further two times, before a final resuspension in 30mL of HEPES wash buffer +2% SDS overnight. The following day, the mixture is briefly heated at 75°C for 15min to dissolve any precipitated SDS, before centrifugation for 1h at room temperature at 4000rcf. The pellet was then washed thrice in 30mL milli-Q water, before transferring to a clean microcentrifuge tube. A further three washes in 1mL of milli-Q water with centrifugation for 15min at 20,000rcf is performed, before the pellet is solubilised in 1mL of 20 mM Na-carbonate buffer pH 10 at 75°C for 30min. Successful purification of S-layer proteins was checked by running on an SDS-PAGE gel and staining with InstantBlue Coomassie Protein Stain (Abcam, ab119211). Concentration of purified proteins was estimated via spectrophotometric quantitation at 280nm and calculated using Beer–Lambert law, assuming SlaA with a theoretical molar extinction coefficient (10) of 1.175 as the major component of the solution. Further validation of purified proteins was also performed via mass spectrometry analysis of the solution during the initial optimization of the purification protocol, which confirmed the presence of SlaA and SlaB.

ConA pull-down and Western blotting

25mL of OD_{595} 0.2 cultures of MW001, SlaA-HA and HA-SlaB cells were collected via centrifugation at 10,000rcf. Cell pellets were resuspended in 300µL of IP lysis buffer (25mM HEPES pH 7.5, 10mM KCL, 1mM $CaCl_2$, 1mM MnCl2) supplemented with 1x RIPA Lysis buffer (Merck, 20-188) and 1x cOmplete protease inhibitor (Roche, 11836170001). The cells were next lysed in a Bioruptor Plus sonication device at 4°C for 7 cycles of 30s on and 30s off, at low power setting. Lysed cells were then centrifuged for 5min at 10,000rcf at 4°C to pellet cellular debris. 200µL of the supernatant were transferred to fresh microcentrifuge tubes and diluted with 300µL of IP lysis buffer. 80µL of the remaining supernatant of each sample was transferred to a fresh tube and 20µL of 1x NuPAGE LDS sample buffer (Thermo Fisher Scientific, NP0007) containing 10% β-mercaptoethanol added, and set aside for use as cell lysate control.

60µL of magnetic agarose concanavalin A beads (Antibodies-online, ABIN6952467) were equilibrated by washing thrice in 1mL of IP lysis buffer on a magnetic rack. Pre-equilibrated beads were resuspended in 90µL IP lysis buffer and 30µL added to each prepared supernatant earlier. ConA pull-down was performed on a rotating mixer at 4°C overnight. Following that, the supernatant was discarded and beads washed thrice with 1mL of ice-cold IP lysis buffer for

5min on the rotating mixer at 4°C. Next, the beads were resuspended in 60µL of 1x NuPAGE LDS sample buffer (Thermo Fisher Scientific, NP0007) containing 10% β-mercaptoethanol. Cell lysate control and ConA pull-down samples were incubated at 98°C for 5min. Following a brief centrifugation, the samples were loaded and ran on a NuPAGE 4 to 12% Bis-Tris gels (Invitrogen) at a constant 150V for 60-80min using MOPS running buffer. Proteins were transferred to nitrocellulose membranes at a constant 100V at 4°C for 60min. Blocking was performed using 5% milk in PBST for 1h, followed by incubation with primary antibodies (Supplementary Table S2) in PBST +5% milk overnight at 4°C with gentle agitation. The membrane was washed in PBST thrice for 5min and then incubated with PBST +5% milk containing IRDye secondary antibodies (Supplementary Table S2) for 2h. Following three washes in PBST, the membrane was imaged using a Bio-Rad ChemiDoc system.

<u>Mass spectrometry</u>

ConA pull-down of *S. acidocaldarius* wild-type DSM639 whole cell lysate was performed using the protocol described above. ConA beads were resuspended in 50µL of 25mM AMBIC 0.1% RapiGest and digested for protein identification by mass spectrometry. Cysteines were reduced by addition of 5µL of a 11.1mg/mL DTT solution to achieve a final concentration of 4mM, and mixed via vortexing before incubating for 10min at 60°C. Alkylation was next performed by addition of 5µL of iodoacetamide (46.6mg/mL) to achieve a final concentration of 14mM, and incubated at room temperature in the dark for 30min. 1µg of trypsin was added for in-bead digestion for 3h at 37°C at 450rpm shaking on a Eppendorf thermomixer. Beads were removed and digestion performed overnight at 37°C. The following day, 1µL of trifluoracetic acid was added to a final concentration of 0.5% (v/v) and incubated for 45min at 37°C to stop digestion and induce RapiGest degradation. The mixture was centrifuged for 15min at 13,000rcf at 4°C. The supernatant was transferred to a fresh microcentrifuge tube and 5µL injected over a 60min programme for mass spectrometry analysis.

LC-MS/MS was performed on an Ultimate U3000 HPLC (ThermoFisher Scientific) hyphenated to an Orbitrap QExactive Classic mass spectrometer (ThermoFisher Scientific). Peptides were trapped on a C18 Acclaim PepMap 100 (5µm, 300µm x 5mm) trap column (ThermoFisher Scientific) and eluted onto an IonOpticks Aurora Ultimate column (1.7µm, 75µm x 250mm) using 52 minute gradient of acetonitrile (7 to 40%). For data dependent acquisition, MS1 scans were acquired at a resolution of 70,000 (AGC target of 1e6 ions with a maximum injection time of 65ms) followed by ten MS2 scans acquired at a resolution of 17,500 (AGC target of 2e5 ions with a maximum injection time of 100ms) using a collision induced dissociation energy of 25. Dynamic exclusion of fragmented m/z values was set to 40s.

Raw data were imported and processed in Proteome Discoverer v3.1 (Thermo Fisher Scientific). The raw files were submitted to a iterative database search using Proteome Discoverer with Sequest HT and Inferis Rescoring against the UniProt reference proteome for *S. acidocaldarius* with methionine oxidation and cysteine carbamidomethylation set as variable and fixed modifications, respectively. The spectra identification was performed with the following parameters: MS accuracy of 10ppm MS/MS accuracy of 0.02Da; up to two trypsin missed cleavage sites were allowed. Only rank 1 peptide identifications of high confidence (FDR<1%) were accepted.

<u>Trichloroacetic acid precipitation of SlaA from growth medium</u>

S. acidocaldarius cells were grown to an OD_{595} of 0.5 under standard culturing conditions. 20mL of each culture was centrifuged at 4000rcf for 10min to pellet the cells. 15mL of the supernatant is then removed carefully, and passed through a 0.22µm syringe filter into a fresh tube to remove all remaining cells.

555µL of 100% (w/v) trichloroacetic acid (Sigma-Aldrich, T6399) was added to 5mL of the filtered media to obtain a final concentration of 10% (v/v) and the mixture incubated on ice for 1h with intermittent mixing. Precipitated proteins were pelleted via centrifugation at 20,000rcf at 4°C for 10min. The pellet is next washed in 1mL of ice-cold acetone, and pelleted again at 20,000rcf at 4°C for 10min. The acetone was discarded and the pellet dried in a Eppendorf Vacufuge plus vacuum concentrator for 5min at room temperature. Precipitated proteins were then resuspended in 50µL of 1x NuPAGE LDS sample buffer (Thermo Fisher Scientific,

NP0007) containing 10% β-mercaptoethanol, and incubated at 98°C for 5min. 20µL of each sample was loaded and ran on a NuPAGE 4 to 12% Bis-Tris gels (Invitrogen, NP0321BOX) at a constant 150V for 60-80min using MOPS running buffer. Proteins were visualised via staining of the gel with InstantBlue Coomassie Protein Stain (Abcam, ab119211).

Live cell microscopy

Live cell imaging was performed using the Sulfoscope set-up as described previously (11–13). Briefly, Attofluor chambers (Invitrogen, A7816) were assembled with 25mm coverslips and filled with 300µL Brock media supplemented with 0.1% (w/v) NZ-amine and 0.2% (w/v) sucrose. The media was allowed to dry onto the surface of the coverslip at 75˚C before the chambers were rinsed twice with fresh Brock media and placed into the pre-heated Sulfoscope. The setup was allowed to equilibrate to 75°C. 5mL of *S. acidocaldarius* cell culture at an OD_{595} of 0.15-0.30 was stained with 1:5000 CellMask Deep Red Plasma Membrane (Invitrogen, C10046) and kept at 75°C in a polystyrene box with pre-warmed metallic beads (Gibco Lab Armor, 10120988).

Gelrite-Brock medium pads were prepared by cutting from a petri dish with a 7mm diameter circle punch and placed onto 13mm circular coverslips. Pads were prewarmed for 5min at 75°C in a bead bath prior to imaging. 400µL of the stained cell suspension was then transferred to the pre-heated Attofluor chamber and immobilised using Gelrite-Brock medium pads with the concave edges of the pad in the middle of the imaging chamber. Imaging was performed at this concave edge where diffusion is limited but cells are not subjected to mechanical stress by the pad.

Images were acquired as described above, with a 60× oil immersion objective (Plan Apo 60×/1.45, Nikon) using a custom formulated immersion oil for high temperature imaging (maximum refractive index matching at 70°C, n=1.515 ±0.0005; Cargille Laboratories). Images were acquired at intervals of 15s for 2.5h or until any cell death was observed. XY drift was corrected after acquisition using the ImageJ plugin StackReg (14).

Supplementary Tables

Supplementary Table S1: Strains used in this study

Genotype	Strain	Figures
Δ*pyrEF*	MW001	1A-C, 3A-B, 4A, 4D-G, S1C, S1G, S2A-D, S2F, S4A-C
Δ*pyrEF* Δ*slaA*	SF40	1A-C, 2F, 3A-B, 4B, 4D-G, S1A, S1G, S2F, S4A-C
Δ*pyrEF* Δ*slaB*	SF69	1A-C, 3A-B, 4C-E, S1A, S1G, S2F, S4A-C
Δ*pyrEF* Δ*slaAB*	SF101	1A-C, 3A-B, S1A, S1G, S2F, S4A-B
Δ*pyrEF* Δ*saci1846*	SF154	1C, S1A, S1F, S1G, S2F
Δ*pyrEF* Δ*slaB* Δ*saci1846*	SF146	1C, S1A, S1G, S2F
Δ*pyrEF* pSVA-promoter^{ara}:SlaA-HA	SF57	2A, 2E-F, S1C
Δ*pyrEF* Δ*slaA* pSVA-promoter^{ara}:SlaA-HA	SF58	2A, 2C-D, 2F
Δ*pyrEF* pSVA-promoter^{ara}:Vps4^{E209Q}-His$_6$	SF93	3C, 3G, S3A-B, S3H
Δ*pyrEF* Δ*slaA* pSVA-promoter^{ara}:Vps4^{E209Q}-His$_6$	SF85	3C-F, S3A-E, S3G
Δ*pyrEF* Δ*slaB* pSVA-promoter^{ara}:Vps4^{E209Q}-His$_6$	SF86	3G, S3A-B, S3H
Δ*pyrEF* Δ*slaAB* pSVA-promoter^{ara}:Vps4^{E209Q}-His$_6$	SF130	S3A-B
Wild-type	DSM639	S1B, S2E
Δ*pyrEF* pSVA-promoter^{ara}:HA-SlaB	SF72	S1C, S3F
Δ*pyrEF* Δ*slaA* pSVA-promoter^{ara}:HA-SlaB	SF148	S3F

Supplementary Table 2: Antibodies used in this study

Antibody	Host organism	Dilution	Catalog number
Anti-HA IgG	Mouse	1:1000	Invitrogen, 261831MG
Anti-CdvB serum	Mouse	1:1000	Lab collection, see (15)
Anti-CdvB1 IgY	Chicken	1:1000	Lab collection, see (15)
Anti-CdvB2 peptide Ab	Guinea pig	1:1000	Lab collection, see (15)
Anti-Mouse IgG, AF488	Goat	1:1000	Invitrogen, A11034
Anti-Chicken IgY, AF546	Goat	1:1000	Invitrogen, A11040
Anti-Guinea pig IgG, AF647	Goat	1:1000	Invitrogen, A21450
Anti-Mouse IgG IRDye 800CW	Goat	1:10,000	LI-COR Bio, 926-32210

References

1. T. D. Brock, K. M. Brock, R. T. Belly, R. L. Weiss, Sulfolobus: a new genus of sulfur-oxidizing bacteria living at low pH and high temperature. *Arch Mikrobiol* **84**, 54–68 (1972).

2. M. Wagner, *et al.*, Versatile Genetic Tool Box for the Crenarchaeote Sulfolobus acidocaldarius. *Front Microbiol* **3**, 214 (2012).

3. D. N. Mastronarde, SerialEM: A Program for Automated Tilt Series Acquisition on Tecnai Microscopes Using Prediction of Specimen Position. *Microscopy and Microanalysis* **9**, 1182–1183 (2003).

4. W. J. H. Hagen, W. Wan, J. A. G. Briggs, Implementation of a cryo-electron tomography tilt-scheme optimized for high resolution subtomogram averaging. *Journal of Structural Biology* **197**, 191–198 (2017).

5. J. Zivanov, *et al.*, New tools for automated high-resolution cryo-EM structure determination in RELION-3. *Elife* **7**, e42166 (2018).

6. S. Zheng, *et al.*, AreTomo: An integrated software package for automated marker-free, motion-corrected cryo-electron tomographic alignment and reconstruction. *Journal of Structural Biology: X* **6**, 100068 (2022).

7. A. Burt, *et al.*, An image processing pipeline for electron cryo-tomography in RELION-5. *FEBS Open Bio* **14**, 1788–1804 (2024).

8. L. Gambelli, *et al.*, Structure of the two-component S-layer of the archaeon Sulfolobus acidocaldarius. *eLife* **13**, e84617 (2024).

9. P. Simonin, C. Lombard, A. Huguet, A. Kish, Improved Isolation of SlaA and SlaB S-layer proteins in Sulfolobus acidocaldarius. *Extremophiles* **24**, 673–680 (2020).

10. S. C. Gill, P. H. von Hippel, Calculation of protein extinction coefficients from amino acid sequence data. *Anal Biochem* **182**, 319–326 (1989).

11. F. Hurtig, *et al.*, The patterned assembly and stepwise Vps4-mediated disassembly of composite ESCRT-III polymers drives archaeal cell division. *Sci Adv* **9**, eade5224 (2023).

12. A. A. Pulschen, *et al.*, Live Imaging of a Hyperthermophilic Archaeon Reveals Distinct Roles for Two ESCRT-III Homologs in Ensuring a Robust and Symmetric Division. *Current Biology* **30**, 2852-2859.e4 (2020).

13. A. Cezanne, B. Hoogenberg, B. Baum, Probing archaeal cell biology: exploring the use of dyes in the imaging of Sulfolobus cells. *Front Microbiol* **14**, 1233032 (2023).

14. P. Thévenaz, U. E. Ruttimann, M. Unser, A pyramid approach to subpixel registration based on intensity. *IEEE Trans Image Process* **7**, 27–41 (1998).

15. G. Tarrason Risa, *et al.*, The proteasome controls ESCRT-III-mediated cell division in an archaeon. *Science* **369**, eaaz2532 (2020).
